# Safety and immunogenicity of ChAdOx1 85A prime followed by MVA85A boost compared with BCG revaccination among Ugandan adolescents who received BCG at birth: a randomised, open-label trial

**DOI:** 10.1016/S1473-3099(23)00501-7

**Published:** 2024-03

**Authors:** Anne Wajja, Beatrice Nassanga, Agnes Natukunda, Joel Serubanja, Josephine Tumusiime, Helen Akurut, Gloria Oduru, Jacent Nassuuna, Joyce Kabagenyi, Hazel Morrison, Hannah Scott, Rebecca Powell Doherty, Julia L Marshall, Ingrid Cabrera Puig, Stephen Cose, Pontiano Kaleebu, Emily L Webb, Iman Satti, Helen McShane, Alison M Elliott, TB042 Study Team, Milly Namutebi, Esther Nakazibwe, Caroline Onen, Barbara Apuule, Florence Akello, Mike Mukasa, Marble Nnaluwooza, Moses Sewankambo, Sam Kiwanuka, Fred Kiwudhu, Esther Imede, Gyaviira Nkurunungi, Prossy Kabuubi Nakawungu, Grace Kabami, Emmanuel Nuwagaba, Mirriam Akello

**Affiliations:** a MRC/UVRI and LSHTM Uganda Research Unit, Entebbe, Uganda; b Department of Global Health, Amsterdam University Medical Centers, Amsterdam, Netherlands; c Amsterdam Institute for Global Health and Development, Amsterdam University Medical Centers, Amsterdam, Netherlands; d Department of Clinical Research, London School of Hygiene & Tropical Medicine, London, UK; e Department of Infectious Disease Epidemiology, London School of Hygiene & Tropical Medicine, London, UK; f The Jenner Institute, Old Road Campus Research Building, University of Oxford, Oxford, UK; g Department of Immunology and Molecular Biology, School of Biomedical Sciences, College of Health Sciences, Makerere University, Kampala, Uganda; h Centre for Clinical Vaccinology and Tropical Medicine, The Jenner Institute, University of Oxford, Churchill Hospital, Oxford, UK

## Abstract

**Background:**

BCG confers reduced, variable protection against pulmonary tuberculosis. A more effective vaccine is needed. We evaluated the safety and immunogenicity of candidate regimen ChAdOx1 85A–MVA85A compared with BCG revaccination among Ugandan adolescents.

**Methods:**

After ChAdOx1 85A dose escalation and age de-escalation, we did a randomised open-label phase 2a trial among healthy adolescents aged 12–17 years, who were BCG vaccinated at birth, without evident tuberculosis exposure, in Entebbe, Uganda. Participants were randomly assigned (1:1) using a block size of 6, to ChAdOx1 85A followed by MVA85A (on day 56) or BCG (Moscow strain). Laboratory staff were masked to group assignment. Primary outcomes were solicited and unsolicited adverse events (AEs) up to day 28 and serious adverse events (SAEs) throughout the trial; and IFN-γ ELISpot response to antigen 85A (day 63 [geometric mean] and days 0–224 [area under the curve; AUC).

**Findings:**

Six adults (group 1, n=3; group 2, n=3) and six adolescents (group 3, n=3; group 4, n=3) were enrolled in the ChAdOx1 85A-only dose-escalation and age de-escalation studies (July to August, 2019). In the phase 2a trial, 60 adolescents were randomly assigned to ChAdOx1 85A–MVA85A (group 5, n=30) or BCG (group 6, n=30; December, 2019, to October, 2020). All 60 participants from groups 5 and 6 were included in the safety analysis, with 28 of 30 from group 5 (ChAdOx1 85A–MVA85A) and 29 of 30 from group 6 (BCG revaccination) analysed for immunogenicity outcomes. In the randomised trial, 60 AEs were reported among 23 (77%) of 30 participants following ChAdOx1 85A–MVA85A, 31 were systemic, with one severe event that occurred after the MVA85A boost that was rapidly self-limiting. All 30 participants in the BCG revaccination group reported at least one mild to moderate solicited AE; most were local reactions. There were no SAEs in either group. Ag85A-specific IFN-γ ELISpot responses peaked on day 63 in the ChAdOx1 85A–MVA85A group and were higher in the ChAdOx1 85A-MVA85A group compared with the BCG revaccination group (geometric mean ratio 30·59 [95% CI 17·46–53·59], p<0·0001, day 63; AUC mean difference 57 091 [95% CI 40 524–73 658], p<0·0001, days 0–224).

**Interpretation:**

The ChAdOx1 85A–MVA85A regimen was safe and induced stronger Ag85A-specific responses than BCG revaccination. Our findings support further development of booster tuberculosis vaccines.

**Funding:**

UK Research and Innovations and Medical Research Council.

**Translations:**

For the Swahili and Luganda translations of the abstract see Supplementary Materials section.

## Introduction

Tuberculosis, temporarily second to SARS-CoV-2 as an infectious cause of death,^1^ is likely to regain its position as “captain of all these men of death”^2^ as COVID-19 mortality declines. Contrasting the rapid COVID-19 response, milestones in tuberculosis control have occurred across centuries, beginning with identification of the pathogen in 1882, development of the BCG vaccine (the only licenced vaccine) in the 1920s,^3^ and development of effective chemotherapy in the mid-20th century. Despite worldwide neonatal BCG vaccination, about 1·6 million people died of tuberculosis in 2021.^1^

An effective vaccine would revolutionise tuberculosis control. Neonatal BCG vaccination protects against disseminated infant disease,^4^ but efficacy against pulmonary disease, which peaks in adolescents and young adults,^5^ declines with time^6^ and varies with latitude, from over 80% efficacy in temperate countries to zero close to the equator.^7^ This inadequate protection is crucial since smear-positive pulmonary tuberculosis drives transmission. Effective boosting for adolescents has great potential as a cost-effective intervention.^8^ Evidence suggesting protection against *Mycobacterium tuberculosis* infection following BCG revaccination reopened debate on revaccination in adolescents,^9^ but it is likely that efficacy will vary geographically, and be lowest in low-income equatorial environments.^10^, ^11^ A more effective booster strategy might be needed for such settings.

**Research in context**

**Evidence before this study**

Between April 25, 2017, and March 30, 2023, we searched PubMed using the medical subject headings “ChAdOx1 85A” AND “MVA85A” AND “BCG” OR “clinical trial”. There were no language restrictions. We identified one published clinical trial on ChAdOx1 85A–MVA85A, which had been done in healthy BCG-vaccinated UK adults. Although this clinical trial showed the safety and immunogenicity of the ChAdOx1 85A–MVA85A regimen, it did not include a BCG revaccination comparison group. Moreover, the clinical trial was done in a non-tuberculosis endemic area and in a temperate region where tuberculosis vaccines, including BCG and MVA85A, have been observed to induce superior protection and immune responses than in tropical regions.

Studies done among adolescents living in high tuberculosis transmission areas in South Africa have shown the potential effectiveness and cost-effectiveness of BCG revaccination at preventing pulmonary tuberculosis, and that it has significantly higher efficacy than placebo at preventing sustained *Mycobacterium tuberculosis-*specific IFN-γ release assay conversion, suggesting protection against infection. Despite these promising findings, South Africa is a temperate region and the effects of BCG revaccination have been variable in other settings—for example in Brazil, ranging from 0% in tropical Manaus to 19% in coastal Salvador. Revaccination strategies need to be investigated across the range of environments in which they might be implemented.

Therefore, we undertook a proof-of-concept study to evaluate the comparative immunogenicity of subunit boosting with ChAdOx1 85A–MVA85A compared with BCG revaccination in adolescents residing in a tuberculosis-endemic region in the tropics.

**Added value of this study**

This was the first clinical trial to evaluate safety and immunogenicity of a ChAdOx1 85A–MVA85A boost following BCG vaccination at birth, and to compare it with BCG revaccination, among adolescents residing in a tuberculosis-endemic area in the tropics. The trial was done among healthy participants in an established birth cohort who had documented BCG vaccination at birth with a known vaccine strain (BCG Moscow), and with a data archive of lifetime infectious exposures that might influence vaccine responses. In this study, we show that the ChAdOx1 85A–MVA85A boost is safe in the Ugandan population and that it induces superior cellular and humoral immune responses to antigen 85A (the vaccine antigen, also present in BCG) compared with BCG revaccination. Additional exploratory analysis shows that ChAdOx1 85A–MVA85A induces similar immune responses in Ugandan and UK populations.

**Implications of all the available evidence**

This study shows that ChAdOx1 85A–MVA85A induces superior immunogenicity to antigen 85A compared with BCG revaccination. This trial was not powered to evaluate the efficacy of ChAdOx1 85A–MVA85A versus BCG revaccination at preventing *M tuberculosis* infection or disease. In-vitro studies evaluating trial samples using mycobacteria growth inhibition assays are ongoing to investigate functionality of the induced immune responses further. Exploratory analysis is also ongoing to investigate the effect of infectious exposures to date on the immune response to the two-vaccine regimens.

Vaccines based on live, non-replicating viral vectors are safe and effective.^12^ Heterologous prime-boost regimens using different vectors expressing the same antigen induce potent T-cell and humoral responses.^13^ Vaccines based on specific tuberculosis antigens might provide superior boosting, compared with BCG revaccination.^14^ While tuberculosis vaccine development remains hampered by imperfect understanding of correlates of protection, there is increasing evidence of a role for antibodies; thus, vaccines that induce strong humoral and cellular immunity might provide protection superior to that offered by the BCG vaccine, where T-cell responses dominate.^15^ A combination of viral-vectored vaccines expressing the immunogenic and immunodominant secretory tuberculosis antigen 85A (ChAdOx1 85A prime, followed by MVA85A boost [ChAdOx1 85A–MVA85A]) induced both T-cell and B-cell responses and, given after the BCG vaccine, was more effective than BCG vaccination alone in a mouse model,^16^ and safe and immunogenic in healthy adults in the UK.^17^ Therefore, we hypothesised that ChAdOx1 85A–MVA85A would induce stronger T-cell and B-cell responses among Ugandan adolescents, a low-income equatorial country, who received the BCG vaccine at birth (analogous to the triple vaccine regimen in the mouse model) than could be achieved by BCG revaccination in this age group.

Since ChAdOx1 85A (unlike MVA85A^18^) had not been tested in Ugandan adolescents, we first undertook ChAdOx1 85A dose escalation and age de-escalation in Uganda. We then did an open-label phase 2a trial among Ugandan adolescents participating in the Entebbe Mother and Baby Study (EMaBS) birth cohort^19^ who received the BCG vaccine at birth with a documented vaccine strain, to compare ChAdOx1 85A–MVA85A safety and immunogenicity versus BCG revaccination. The availability of the good manufacturing product material for both ChAdOx1 85A and MVA85A meant we could evaluate whether this single-antigen regimen was more immunogenic than BCG revaccination in this important target population, and provide insights towards consideration of multivalent viral-vectored vaccines that are in preclinical development.^20^, ^21^

## Methods

### Study design

We did ChAdOx1 85A dose-escalation and age de-escalation among Ugandan participants, followed by a randomised, open-label phase 2a trial comparing safety and immunogenicity of ChAdOx1 85A–MVA85A with BCG revaccination among Ugandan adolescents who were BCG vaccinated at birth. The study took place at Entebbe Hospital and the Medical Research Council (MRC)/Uganda Virus Research Institute (UVRI) and London School of Hygiene & Tropical Medicine (LSHTM) Uganda Research Unit, Entebbe, Uganda. It was approved by the UVRI Research Ethics Committee (GC/127/17/09/621), Uganda National Council of Science and Technology (HS 2346), Ugandan National Drug Authority (CTA0063), LSHTM Research Ethics Committee (14598), and Oxford Tropical Research Ethics Committee (2–18), in accordance with the principles of the Declaration of Helsinki and International Conference on Harmonisation of Good Clinical Practice guidelines.

### Participants

We enrolled adolescents and their parents or guardians from EMaBS, a birth cohort that was recruited from 2003 to 2006 to investigate the effects of anthelmintic treatment during pregnancy and childhood on infant vaccine responses.^19^ Parents or guardians were enrolled in the ChAdOx1 85A dose escalation study, and adolescents in the ChAdOx1 85A dose-escalation and age de-escalation studies and the open-label trial.

Adolescents were aged 12–17 years, with documented immunisation with the Moscow BCG-I strain from the Serum Institute of India, Pune, India, less than 2 weeks since birth, with written informed assent and parental or guardian consent. Adults were aged 18–49 years, with a BCG scar or a documented previous BCG vaccination, and provided written informed consent. All participants were residents within the study area, agreed to refrain from blood donation and to avoid pregnancy during the trial (if applicable), and were able and willing to comply with study requirements.

Participants were excluded if they had current or previous tuberculosis treatment; lived with a patient with tuberculosis within 6 months to enrolment; received a tuberculin skin test less than 90 days before enrolment; were ELISpot positive for *M tuberculosis* infection, pregnant, or lactating; had a clinically significant history, or evidence of illness (including HIV, hepatitis B or C infection, or malaria); used immunosuppressive agents less than 2 months before enrolment, immunoglobulins or blood products less than 3 months before enrolment, or other live vaccines or investigational products less than 1 month before enrolment; or had history of anaphylaxis to vaccination or allergy likely to be exacerbated by study vaccine components.

### Randomisation and masking

Participants in the randomised trial were assigned (1:1) to receive ChAdOx1 85A–MVA85A or BCG revaccination. The randomisation sequence was generated in Stata version 15.0 by an independent statistician with a block size of six. Eligible volunteers were sequentially allocated to a randomisation number by the screening interviewer. Sealed envelopes, labelled with the randomisation number, indicated which vaccines to give. It was not possible to mask participants or clinic staff to allocation, due to the differences in vaccine administration and schedules. Laboratory staff were masked to allocation for the outcome assays.

### Procedures

ChAdOx1 85A (lot number 02N12-01) was manufactured at the Clinical Biomanufacturing Facility, University of Oxford, Oxford, UK, and MVA85A (lot number 0050811) at IDT Biologika, Dessau-Rosslau, Germany. They were shipped to UVRI on dry ice. The BCG vaccine (Moscow strain 361-I, batch numbers 0378G149, G700, and 037G7200) was manufactured at the Serum Institute of India, and shipped at 2°C to 8°C. Vaccines were stored at the UVRI–International AIDS Vaccine Initiative's HIV Vaccine Program pharmacy in Entebbe, Uganda, at –80°C (ChAdOx1 85A and MVA85A) or 2°C to 8°C (BCG). On vaccination day, vaccines were transported in temperature-monitored credo boxes to the clinic for reconstitution and administration.

ChAdOx1 85A and MVA85A were supplied as liquid formulations, ready to use. BCG vials containing 2–8 × 10^5^ colony-forming units (CFUs) were reconstituted with sodium chloride injection (1 mL), making ten doses per vial. Clinic nurses gave ChAdOx1 85A (different doses) and MVA85A (1 × 10^8^ plaque-forming units [PFUs]) intramuscularly on the left arm^17^ and BCG (0·1 mL) intradermally on the right arm. The injection site was covered with a dressing for 30 min after vaccination. Participants were observed for adverse events (AEs) for 60 min after vaccination.

In adult dose-escalation studies, group 1 (n=3) received ChAdOx1 85A at 5 × 10^9^ viral particles (VPs); and after a safety review, group 2 (n=3) received ChAdOx1 85A at 2·5 × 10^10^ VP. After a review of adult participants' data by the data and safety monitoring board, adolescents received ChAdOx1 85A at 5 × 10^9^ VP (group 3, n=3) and ChAdOx1 85A at 2·5 × 10^10^ VP (group 4, n=3). After a further review by the data and safety monitoring board, 60 adolescents were randomly assigned in the phase 2a trial: 30 (group 5) to ChAdOx1 85A, 2·5 × 10^10^ VP at day 0 followed by MVA85A, 1 × 10^8^ PFUs on day 56, and 30 (group 6) received the BCG vaccine (licensed dose) at day 0. In group 5, timing of the MVA85A boost and subsequent sampling was disrupted by the COVID-19 lockdown such that the time between ChAdOx1 85A prime and MVA85A boost was extended from day 56 to days 284–297 in 21 participants; follow-up visits for group 6 were similarly delayed for 20 participants (henceforth referred to as the delayed group).

Participants from groups 1–4 were followed up on days 2, 14, 28, 56, and 168, and groups 5–6 on days 2, 14, 28, 56 (or day 365 for the delayed group), 63 (day 372, delayed group), 84 (day 393, delayed group), 140 (day 449, delayed group), and 224 (day 533, delayed group).

Safety was assessed by observation (vital signs), solicited and unsolicited AEs, and biochemistry and haematological assessments. Vital signs were measured 30 and 60 min after vaccination; AEs were assessed by reviewing participants diary cards for 14 days and at clinic visits 2, 4, and 28 after ChAdOx1 85A and BCG, and 7 and 28 days after MVA85A. Additional serious adverse events (SAEs) were assessed to the end of the trial through clinic visits at planned timepoints. Biochemistry (ie, sodium, potassium, urea, creatinine, and albumin) and liver function (ie, gamma-glutamyl transferase, alanine aminotransferase, and alkaline phosphatase) were measured with COBAS 6000 (Hitachi High-Technologies Corporation, Roche, Switzerland) at screening and on day 14 for all groups, and additionally on days 56 and 63 for groups 5 and 6. Haematology assessments were done at the same times, and additionally on day 2 for all and days 140 and 224 for groups 5 and 6, with Sysmex XN1000 (Sysmex Corporation, Kobe, Japan). Clinical AE severity was graded mild, moderate, and severe in accordance with protocol-defined criteria. Laboratory AEs were graded 1 if 1·1–2·4 times the limit of normal, 2 if 2·5–4·9 times, and 3 if 5 times or more, per protocol. Causality was assigned by the study clinician following protocol-defined criteria.

Immunogenicity samples were collected at all timepoints. Ex-vivo IFN-γ ELISpots (Mabtech, Nacka Strand, Sweden) were done on fresh peripheral blood mononuclear cells (PBMCs) from all groups on days 0, 14, 28, and 56, and additionally on day 168 for groups 1–4 and days 63, 84, 140, and 224 for groups 5 and 6 (appendix 3 p 2). PBMCs were stimulated with 2 × 10^5^ CFU/mL of the BCG vaccine (Serum Institute of India, batch 0378G14); tuberculin protein purified derivative (PPD) RT23 (AJVaccines, Copenhagen, Denmark), 20 μg/mL; a single pool of antigen 85A peptides (66 15-mer peptides, overlapping by 10 amino acids), 2 μg/mL; pooled ESAT-6 and CFP-10 (15-mer peptides, Peptide Synthetics, Hampshire, UK), 2 μg/ml; 2 international units per 1 PBMC ChAdOx1-GFP (Jenner Institute, Oxford, UK) to determine vaccine vector-specific responses; 10 μg/mL staphylococcal enterotoxin B from *Staphylococcus aureus* or 10 μg/mL phytohemagglutinin plus 50 ng/mL phorbol 12-myristate 13-acetate (Sigma, Gillingham, UK) as positive controls; media-only wells were included as negative controls.

At the same timepoints, ELISA was used to measure Ag85A-specific and PPD-specific IgG plasma antibody levels (appendix 3 p 2). Capture antigens were 5 μg/mL recombinant Ag85A (BEI Resources, Manassas, VA, USA) or 10 μg/mL tuberculin PPD RT23. Assays were run in duplicate and samples were diluted 1:50 for Ag85A-specific IgG and 1:100 for PPD-specific IgG. A positive control of pooled plasma from active tuberculosis patients was included on each plate to test plate to plate variability. Detection antibody was polyclonal anti-human IgG horseradish peroxidase (Dako, Glostrup, Denmark), developed with o-phenylenediamine (Sigma-Aldrich, St Louis, MO, USA), and stopped after 15 min with 2M sulphuric acid. Optical density was measured at 490 nm test and 630 nm reference wavelength (BioTek Instruments, Winooski, VT, USA). Optical densities for Ag85A-specific and PPD-specific IgG responses in test samples were obtained by subtracting mean optical densities of duplicate blank wells from mean optical densities of duplicate test samples.

Participants were examined for malaria with RT-PCR (appendix 3 pp 2–3)^18^ at screening, and malaria antigen P.f/Pan Rapid Diagnostic Tests (Standard Diagnostics, Davis, CA, USA) at day 0; *Mansonella perstans* by the modified Knotts method^18^ at day 0; and helminths with RT-PCR^18^ at screening, and with the Kato Katz method^18^ at day 0. The trial is registered with ClinicalTrials.gov, NCT03681860.

### Outcomes

Primary safety outcomes were solicited and unsolicited AEs up to day 28 after vaccination, and SAEs throughout the trial. The primary immunogenicity outcome was Ag85A-specific IFN-γ ELISpot response (spot-forming counts [SFC] per 1×10^6^ PBMCs) on day 14 for groups 1–4, and day 63 for groups 5 and 6, and the area under the curve (AUC; days 0–168 for groups 1–4; days 0–224 for groups 5 and 6). The secondary immunogenicity outcome was anti-85A IgG antibody after vaccination and BCG-specific IFN-γ ELISpot. Tertiary immunogenicity outcomes were PPD-specific IFN-γ ELISpot and IgG antibody responses. In groups 5 and 6, the COVID-19 pandemic lockdown, and the consequent delayed MVA85A boost, delayed outcome assessments at days 63 and 168 to up to days 372 and 499, respectively (corresponding to 7 and 112 days after MVA85A boost for group 5). Additional secondary and tertiary outcomes (including flow cytometric and mycobacterial killing assays) will be reported separately.

### Statistical analysis

12 participants were enrolled for dose escalation and age de-escalation. 60 participants (30 per group) for the randomised trial allowed for more than 80% power to detect a 0·29 log_10_ difference in SCF per 10^6^ PBMC with p<0·05 for the primary outcome on day 63, assuming a standard deviation of 0·4 log_10_ SFC per 10^6^ PBMC in peak Ag85A-specific response: similar to the response difference observed between human adenovirus vectored tuberculosis vaccine candidate Aeras 402 alone (day 14) and with MVA85A boost (day 7),^22^ or between BCG (day 28) and MVA85A alone (day 7) in UK participants,^23^ and hence a reasonable difference to expect between the BCG vaccine and ChAdOx1 85A–MVA85A.

Participant characteristics were summarised by group as frequency (%) for categorical and mean (SD) for continuous variables. Adverse event frequencies were summarised overall and by severity and causality, separately by each group. Reactogenicity events and AEs within 28 days of vaccination were summarised. Geometric means and SDs of IFN-γ ELISpot responses to Ag85A and PPD were summarised on day 14 for groups 1–4. No statistical tests were done to compare responses between dose levels (groups 1–4). IFN-γ ELISpot responses to Ag85A and PPD and IgG levels (optical density) on days 63 and 224 were summarised and compared between group 5 and 6 (ChAdOx1 85A–MVA85A *vs* BCG revaccination) using linear regression of log-transformed values, adjusting for corresponding baseline responses, age, and sex. Effect estimates were back-transformed and presented as geometric mean ratios (GMRs), with 95% CIs and p values. The AUC was used to assess the longitudinal immunogenicity of ChAdOx1 85A–MVA85A and BCG, calculated based on cubic splines for responses on days 0, 2, 14, 28, 56, 63, 84, 140, and 224. All analyses were on the basis of the per-protocol population as prespecified in the statistical analysis plan, using Stata version 15.

### Role of the funding source

The funders of the study had no role in study design, data collection, data analysis, data interpretation, or writing of the report.

## Results

**Figure 1:**
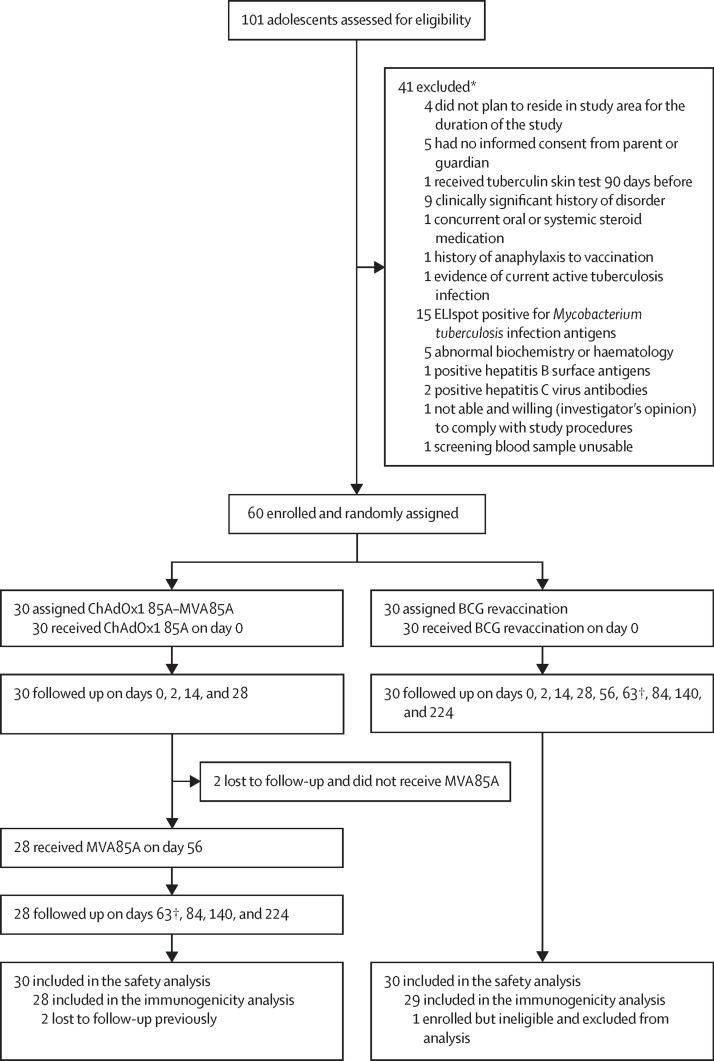
Trial profile Follow-up time points are days 0, 2, 14, 28, 56 (day 365 for delayed group), 63 (day 372 for the delayed group), 84 (day 393 for the delayed gorup), 140 (day 449 for the delayed group), and 224 (day 533 for the delayed group). *Six participants met multiple exclusion criteria. †Primary outcome timepoint.

For dose-escalation and age de-escalation studies, 30 adults and 20 adolescents were screened; six of eight eligible adults and six of 12 eligible adolescents were recruited (July and August, 2019). All completed follow-up. For the phase 2a trial, 101 adolescents were screened; 60 eligible participants were recruited (December, 2019, to October, 2020), with 30 randomly assigned to ChAdOx1 85A–MVA85A (group 5) and 30 to BCG revaccination (group 6). In group 5, seven (23%) of 30 participants received the MVA85A booster as scheduled (median 56 days [IQR 56–57]). However, study activities were then halted by the COVID-19 lockdown in Uganda. When activities resumed, two (7%) of 30 participants in group 5 were lost to follow-up; the remaining 21 (70%) participants received MVA85A boost at a median of 290 days (IQR 286–296) following ChAdOx1 85A vaccination (appendix 3 p 5). Timepoints on day 56 and beyond were correspondingly delayed for 20 (67%) of 30 participants in group 6. Primary endpoints were assessed for participants who completed follow-up (figure 1): 28 participants in group 5 (excluding the two lost to follow-up) and 29 participants in group 6 (one participant positive for latent tuberculosis, erroneously enrolled, was excluded from analysis).

Baseline participant characteristics are shown in table 1. The median age for group 1 was 35 years (IQR 33–38) and for group 2 was 36 years (IQR 35–42). All participants in groups 1 and 2 were female and had BCG scars. The median age for group 3 was 15 years (IQR 15–15) and for group 4 was 15 years (IQR 14–15). 50% of participants in groups 3 and 4 were female and 67% in each group had BCG scars. For groups 5 and 6, baseline characteristics (age, sex, and mean haemoglobin levels) were balanced between the trial groups. The median age was 15 years (IQR 14–16) in both groups. 11 (37%) of 30 participants in group 5 and 13 (43%) of 30 participants in group 6 were female; 22 (73%) in group 5 and 18 (60%) in group 6 had BCG scars. All participants tested negative for malaria by rapid diagnostic tests at enrolment and throughout follow up. Helminth infection was uncommon.

**Table 1:** Baseline demographic characteristics of enrolled participants (groups 1–6)

		**Dose escalation and age de-escalation**				**Randomised trial**	
		Group 1: ChAdOx1 85A at 5 × 10^9^ viral particles (n=3)	Group 2: ChAdOx1 85A at 2·5 × 10^10^ viral particles (n=3)	Group 3: ChAdOx1 85A at 5 × 10^9^ viral particles (n=3)	Group 4: ChAdOx1 85A at 2·5 × 10^10^ viral particles (n=3)	Group 5: ChAdOx1 85A–MVA85A (n=30)	Group 6: BCG revaccination (n=30)
Age, years		34 (33–38)	36 (35–42)	15 (15–15)	15 (14–15)	15 (14–16)	15 (14–16)
Haemoglobin		12·6 (0·83)	14·1 (1·32)	14·4 (0·91)	12·7 (0·55)	13·3 (0·99)	13·5 (1·01)
Sex							
	Male	0	0	2 (67%)	1 (33%)	19 (63%)	17 (57%)
	Female	3 (100%)	3 (100%)	1 (33%)	2 (67%)	11 (37%)	13 (43%)
BCG scar							
	Yes	3 (100%)	3 (100%)	2 (67%)	2 (67%)	22 (73%)	18 (60%)
	No	0	0	1 (33%)	1 (33%)	8 (27%)	12 (40%)
*Schistosoma mansoni* (KK)							
	Uninfected	3 (100%)	3 (100%)	3 (100%)	3 (100%)	28 (93%)	29 (97%)
	Heavy infection (>2000 eggs per g)	0	0	0	0	2 (7%)	1 (3%)
*Schistosoma mansoni* (PCR)							
	Positive	0	1 (33%)	0	0	9 (30%)	7 (23%)
	Negative	3 (100%)	2 (66%)	3 (100%)	3 (100%)	21 (70%)	23 (77%)
Hookworm (KK)							
	Positive	0	0	0	0	0	0
	Negative	3 (100%)	3 (100%)	3 (100%)	3 (100%)	30 (100%)	30 (100%)
Hookworm (PCR)							
	Positive	0	0	0	0	1 (3%)	0
	Negative	3 (100%)	3 (100%)	3 (100%)	3 (100%)	29 (97%)	3 (100%)
*Strongyloides stercolaris* (PCR)							
	Positive	0	0	0	0	0	0
	Negative	3 (100%)	3 (100%)	3 (100%)	3 (100%)	30 (100%)	30 (100%)
*Mansonella perstans*							
	Positive	0	0	0	0	0	0
	Negative	3 (100%)	3 (100%)	3 (100%)	3 (100%)	30 (100%)	30 (100%)
Malaria (PCR)							
	Positive	0	0	0	0	2 (7%)	2 (7%)
	Negative	3 (100%)	3 (100%)	3 (100%)	3 (100%)	28 (93%)	28 (93%)

Data are n (%), median (IQR), or mean (SD). KK=Kato Katz.

The safety outcomes in groups 1–6 up to day 28 are summarised in table 2 (appendix 3 pp 5–7). In the adults' dose-escalation studies (groups 1 and 2), four participants reported 23 solicited AEs (22 among those receiving 5 × 10^9^ VP, one among those receiving 2·5 × 10^10^ VP); of these, eight AEs were local (ie, pain, warmth, or itching at injection site) and 15 systemic (ie, feverishness, fatigue, headache, malaise, arthralgia, or nausea). All reported AEs in these groups were mild to moderate, except for one participant in group 1 who reported severe myalgia, which had resolved by the next clinic visit. One participant per group reported unsolicited AEs, of which none were severe.

**Table 2:** Reactogenicity and adverse events following each vaccination (groups 1–6)

		**Group 1: ChAdOx1 85A at 5 × 10^9^ viral particles (n=3)**	**Group 2: ChAdOx1 85A at 2·5 × 10^10^ viral particles (n=3)**	**Group 3: ChAdOx1 85A at 5 × 10^9^ viral particles (n=3)**	**Group 4: ChAdOx1 85A at 2·5 × 10^10^ viral particles (n=3)**	**Group 5: ChAdOx1 85A (n=30)**	**Group 5: MVA85A (n=28)**	**Group 6: BCG revaccination (n=30)**
**Local adverse events**								
Pain								
	Mild	2 (67%)	1 (33%)	1 (33%)	2 (67%)	8 (27%)	3 (11%)	6 (20%)
	Moderate	1 (33%)	..	..	..	1 (3%)	1 (4%)	..
	Severe	..	..	..	..	..	1 (4%)	..
Warmth								
	Mild	1 (33%)	..	..	..	3 (10%)	..	..
	Moderate	1 (33%)	..	..	..	..	..	..
	Severe	..	..	..	..	..	..	..
Itching								
	Mild	2 (67%)	..	..	..	4 (13%)	1 (4%)	9 (30%)
	Moderate	..	..	..	..	..	..	..
	Severe	..	..	..	..	..	..	..
Swelling								
	Mild	..	..	1 (33%)	1 (33%)	..	1 (4%)	23 (77%)
	Moderate	..	..	..	..	..	1 (4%)	..
	Severe	..	..	..	..	..	..	..
Scaling								
	Mild	..	..	..	..	..	3 (11%)	30 (100%)
	Moderate	..	..	..	..	..	..	..
	Severe	..	..	..	..	..	..	..
**Systemic adverse events**								
Feverishness								
	Mild	2 (67%)	..	..	1 (33%)	3 (10%)	1 (4%)	4 (13%)
	Moderate	1 (33%)	..	..	..	..	..	..
	Severe	..	..	..	1 (33%)	..	1 (4%)	..
Fatigue								
	Mild	2 (67%)	..	..	1 (33%)	2 (7%)	1 (4%)	1 (3%)
	Moderate	..	..	..	1 (33%)	1 (3%)	1 (4%)	..
	Severe	..	..	..	..	..	..	..
Headache								
	Mild	2 (67%)	..	..	2 (67%)	7 (23%)	2 (7%)	5 (17%)
	Moderate	..	..	..	..	..	..	1 (3%)
	Severe	..	..	..	1 (33%)	..	..	..
Malaise								
	Mild	2 (67%)	..	1 (33%)	1 (33%)	1 (3%)	1 (4%)	1 (3%)
	Moderate	..	..	..	..	..	..	..
	Severe	..	..	..	1 (33%)	..	1 (4%)	..
Arthralgia								
	Mild	2 (67%)	..	1 (33%)	1 (33%)	3 (10%)	1 (4%)	1 (3%)
	Moderate	1 (33%)	..	..	..	..	..	..
	Severe	..	..	..	..	..	..	..
Nausea								
	Mild	1 (33%)	..	..	1 (33%)	1 (3%)	..	..
	Moderate	..	..	..	1 (33%)	..	..	..
	Severe	..	..	..	..	..	1 (4%)	..
Myalgia								
	Mild	..	..	..	..	2 (7%)	..	..
	Moderate	..	..	..	..	..	..	..
	Severe	1 (33%)	..	..	..	..	..	..

Data are n (%). Frequency is calculated as the number of volunteers counted once for each adverse event at maximal severity grading following each vaccination.

In the adolescents' dose-escalation studies (groups 3 and 4), 19 solicited AEs were recorded among six adolescents: four AEs among those receiving 5 × 10^9^ VP, and 15 among those receiving 2·5 × 10^10^ VP. Five AEs (ie, mild pain or swelling at injection site) were local and 14 AEs (ie, feverishness, fatigue, headache, malaise, arthralgia, or nausea) were systemic. Most AEs were mild to moderate but one participant in group 4 reported severe feverishness, headache, and malaise, which had resolved by the next visit. One adolescent in group 4 reported an unsolicited AE that was not severe.

In the phase 2a trial, following ChAdOx1 85A vaccination (group 5), 13 (43%) of 30 participants reported at least one solicited AE within 28 days, with 37 different AEs reported (16 local and 21 systemic). Two (7%) of 30 reported at least one unsolicited AE. From subsequent MVA85A vaccination of this group, ten (36%) of 28 reported at least one solicited AE, with 21 different AEs (11 local and 10 systemic), all mild to moderate except for one comprising severe pain at the injection site, fever, nausea, and malaise. In the BCG revaccination group (group 6), all 30 (100%) participants reported at least one solicited AE, all mild to moderate, with local scaling and swelling being the most common; there were 82 different AEs (68 local and 14 systemic) in this group. There were no protocol-defined laboratory AEs and no SAEs for any group.

In groups 1–4, Ag85A-specific and PPD-specific IFN-γ ELISpot responses increased after ChAdOx1 85A vaccination, peaked on day 14, declined by day 168, but remained above baseline levels in all groups (appendix 3 pp 11–12). On day 14, geometric mean (SD) Ag85A specific IFN-γ ELISpot responses were generally lower in adults in group 1 (135·04 [3·22]) and group 2 (141·94 [3·74]) than in adolescents in group 3 (592·97[1·45]) and group 4 (440·64 [1·41]; appendix 3 p 7).

**Figure 2:**
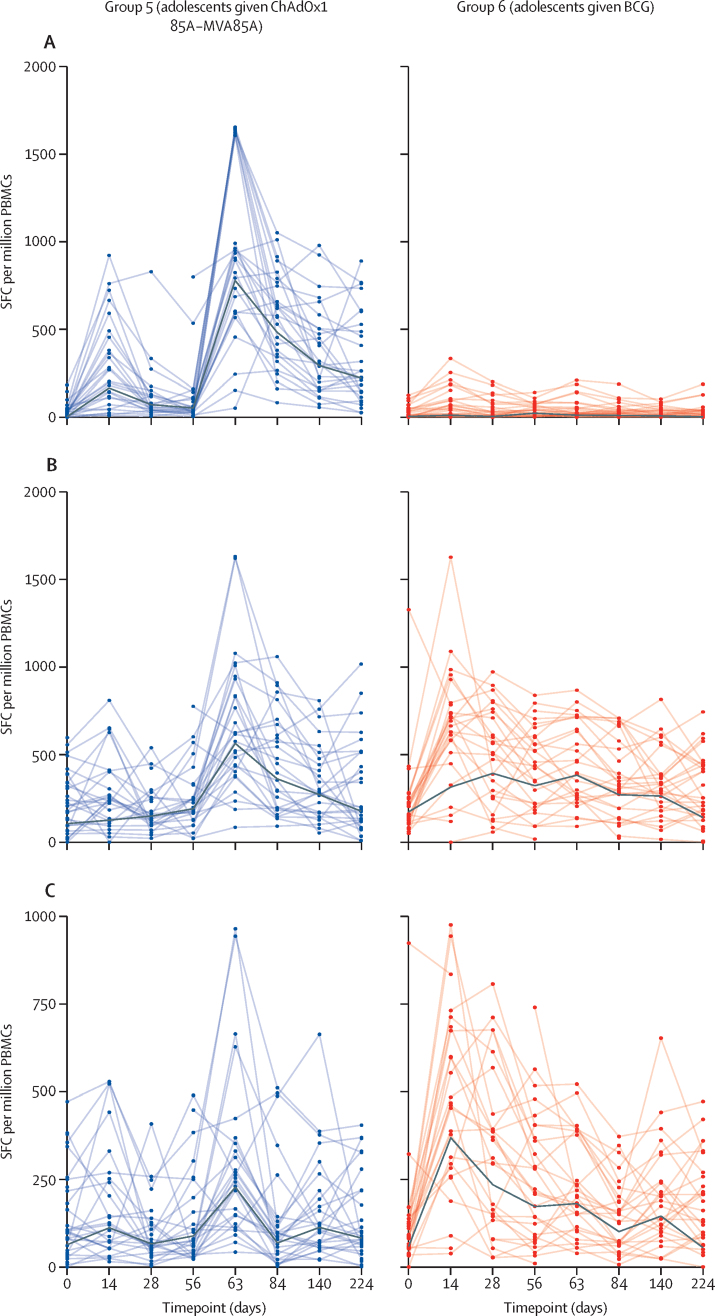
Ex-vivo IFN-γ ELISpot responses to Ag85A, PPD, and BCG in adolescent volunteers vaccinated with ChAdOx1 85A–MVA85A or BCG revaccination (A) Ex-vivo IFN-γ ELISpot responses to Ag85A pool of 66 peptides. (B) Ex-vivo IFN-γ ELISpot responses to PPD. (C) Ex-vivo IFN-γ ELISpot responses to BCG. For all panels, individual values are shown for each volunteer at each follow-up timepoint. The black bold line represents geometric mean. Follow-up timepoints are days 0, 14, 28, 56 (day 365 for the delayed group), 63 (372 for the delayed group), 84 (393 for the delayed group), 140 (449 for the delayed group), and 224 (533 for the delayed group). PBMCs=peripheral blood mononuclear cells. PPD=protein purified derivative. SFCs=spot-forming counts.

In the randomised trial (groups 5 and 6), there were no significant differences in outcomes between the per-protocol and delayed groups (appendix 3 pp 10, 14), so we present immunogenicity results together according to intended sampling times. Ag85A-specific IFN-γ ELISpot responses peaked on day 63 after MVA85A in the ChAdOx1 85A–MVA85A group and on day 14 following BCG revaccination (figure 2). These responses were higher in the ChAdOx1 85A–MVA85A compared with the BCG revaccination group on day 63 (GMR 30·59 [95% CI 17·46 to 53·59], p<0·0001) and throughout follow-up (AUC mean difference 57 091 [95% CI 40 524 to 73 658], p<0·0001; table 3). PPD-specific IFN-γ ELISpot responses peaked on day 63 after MVA85A; after BCG revaccination there was no discrete peak but responses were higher than baseline from day 14 to 140. On day 63, ChAdOx1 85A–MVA85A showed higher PPD-specific IFN-γ ELISpot responses than BCG revaccination (GMR 1·42 [95% CI 1·02 to 1·98], p=0·04), but there was no difference on day 224 (GMR 0·91 [95% CI 0·44 to 1·88], p=0·79) or throughout follow-up (AUC mean difference –15 141 [95% CI –37 060 to 6777], p=0·17, days 0 to 224). ChAdOx1 85A–MVA85A induced significantly lower BCG-specific IFN-γ ELISpot responses than the BCG revaccination group throughout follow-up (AUC mean difference –18 049 [95% CI –32 403 to –3694], p=0·02, days 0 to 224), but no significant difference was observed on day 63 (GMR 1·24 [95% CI 0·83 to 1·86], p=0·29) or on day 224 (GMR 0·80 [95% CI 0·39 to 1·64], p=0·53).

**Table 3:** Differences in IFN-γ ELISpot and IgG responses to Ag85A and PPD between ChAdOx1 85A–MVA85A and BCG revaccination participants at day 63 and area under the curve for days 0 to 224 (groups 5 and 6)

	**AUC (0–224 days)**			**Day 63 unadjusted**				Day 63 adjusted*			**Day 224 unadjusted**				Day 224 adjusted*		
	n	Mean difference, AUC (95% CI)	p value	n	GM (SE)†	GMR (95% CI)	p value	n	GMR (95% CI)	p value	n	GM (SE)†	GMR (95% CI)	p value	n	GMR (95% CI)	p value
**IFN-γ-Ag85a**																	
ChAdOx1 85A–MVA85A	30	57 091 (40 524 to 73 657)	<0·0001	27	779·30 (1·17)	31·76 (17·31 to 57·54)	<0·0001	27	30·59 (17·46 to 53·59)	<0·0001	28	223·56 (1·20)	15·87 (7·91 to 31·84)	<0·0001	28	15·06 (8·15 to 27·84)	<0·0001
BCG revaccination	29	1 (ref)	..	27	24·54 (1·30)	1 (ref)	..	26	1 (ref)	..	29	14·08 (1·34)	1 (ref)	..	28	1 (ref)	..
**IFN-γ-PPD**																	
ChAdOx1 85A–MVA85A	30	−15 141 (−37 060 to 6777)	0·17	27	565·04 (1·13)	1·48 (1·05 to 2·08)	0·027	27	1·42 (1·02 to 1·98)	0·036	28	179·45 (1·26)	0·92 (0·46 to 1·84)	0·808	28	0·91 (0·44 to 1·88)	0·79
BCG revaccination	29	1 (ref)	..	27	382·97 (1·13)	1 (ref)	..	26	1 (ref)	..	29	195·26 (1·29)	1 (ref)	..	28	1 (ref)	..
**IgG-Ag85a**																	
ChAdOx1 85A–MVA85A	30	116 (66 to 166)	<0·0001	28	2·04 (1·06)	1·51 (1·31 to 1·75)	<0·0001	28	1·50 (1·32 to 1·70)	<0·0001	28	1·67 (1·05)	1·28 (1·13 to 1·45)	<0·0001	28	1·26 (1·15 to 1·39)	<0·0001
BCG revaccination	29	1 (ref)	..	29	1·35 (1·05)	1 (ref)	..	29	1 (ref)	..	29	1·31 (1·04)	1 (ref)	..	29	1 (ref)	..
**IgG-PPD**																	
ChAdOx1 85A–MVA85A	30	−21 (−63 to 20)	0·31	28	1·79 (1·04)	1·00 (0·90 to 1·12)	0·95	28	0·96 (0·89 to 1·04)	0·32	28	1·67 (1·03)	0·98 (0·88 to 1·08)	0·651	28	0·93 (0·87 to 1·00)	0·04
BCG revaccination	29	1 (ref)	..	29	1·78 (1·04)	1 (ref)	..	29	1 (ref)	..	29	1·71 (1·04)	1 (ref)	..	29	1 (ref)	..
**IFN-γ-BCG**																	
ChAdOx1 85A–MVA85A	29	−18 049 (−32 403 to −3694)	0·02	27	228·63 (1·16)	1·25 (0·84 to 1·88)	0·27	27	1·24 (0·83 to 1·86)	0·29	28	85·87 (1·26)	0·82 (0·40 to 1·69)	0·58	28	0·80 (0·39 to 1·64)	0·53
BCG revaccination	29	1 (ref)	..	27	182·25 (1·15)	1 (ref)	..	26	1 (ref)	..	29	104·68 (1·32)	1 (ref)	..	28	1 (ref)	..

AUC=area under the curve. GM=geometric mean. GMR=geometric mean ratio. PPD=protein purified derivative.

* Adjusted for corresponding baseline responses, sex, and age.

† Geometric mean of outcome +1.

**Figure 3:**
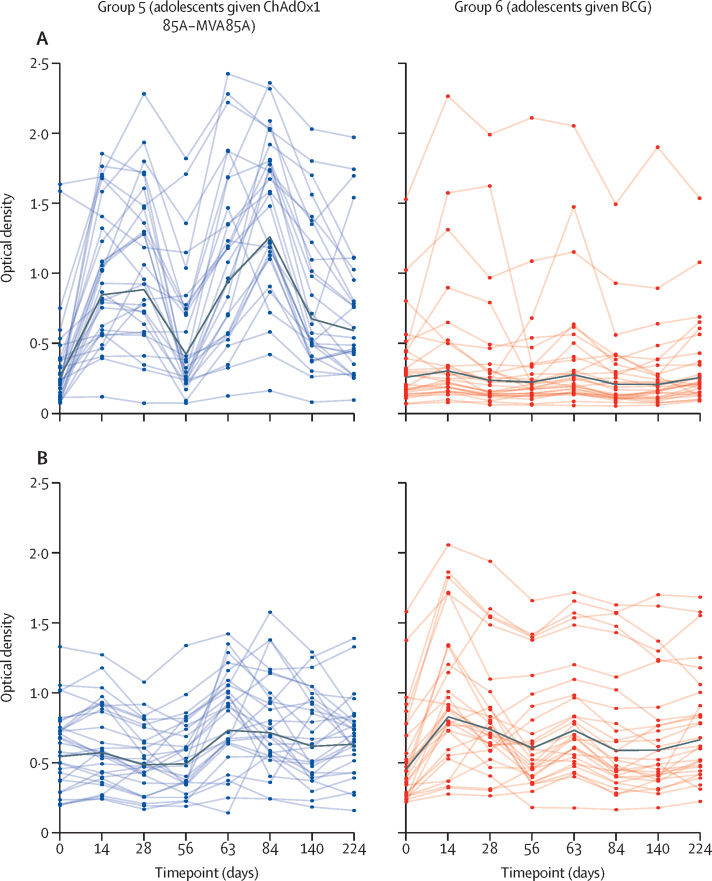
Plasma IgG responses to Ag85A and PPD in adolescent volunteers vaccinated with ChAdOx1 85A–MVA85A or BCG revaccination (A) Plasma IgG responses to recombinant Ag85A. (B) Plasma IgG responses to PPD. For all panels, individual values are shown for each volunteer at each follow-up timepoint. The black bold line represents geometric mean. Follow-up timepoints are days 0, 14, 28, 56 (day 365 for the delayed group), 63 (372 for the delayed group), 84 (393 for the delayed group), 140 (449 for the delayed group), and 224 (533 for the delayed group). PPD=protein purified derivative.

Ag85A-specific and PPD-specific IgG responses for groups 1–6 are shown in figure 3 (appendix 3 p 13). Participants in the dose-escalation and age de-escalation groups (groups 1–4) had increased Ag85A-specific (but not PPD-specific) IgG responses after vaccination with ChAdOx1 85A (appendix 3 p 7). In the randomised trial detailed in table 3, the ChAdOx1 85A–MVA85A group (group 5) had higher Ag85A-specific IgG responses on day 63 than the BCG revaccination group (group 6; GMR 1·50 [95% CI 1·32 to 1·70], p<0·0001). These differences persisted to day 224 (GMR 1·26 [95% CI 1·15 to 1·39], p<0·0001) and throughout follow-up (AUC mean difference 116 [95% CI 66 to 166], p<0·001, days 0 to 224). For PPD-specific IgG, there were no significant differences in AUC between groups 5 and 6 (AUC mean difference –21 [95% CI –63 to 20], p=0·31, days 0–224); higher responses were seen on day 224 within the BCG revaccination group (GMR 0·93 [95% CI 0·87 to 1·0], p=0·04) but not on day 63 (GMR 0·96 [95% CI 0·89 to 1·04], p=0·32).

We observed modest correlations between vaccine-specific IFN-γ ELISpot and IgG antibody responses on day 63 and 224 in groups 5 and 6 (appendix 3 pp 7–8). In group 5, we observed very low correlation between Ag85A-specific IFN-γ ELISpot responses on day 28 following ChAdOx1 85A vaccination and peak IFN-γ ELISpot responses on day 63 (Pearson's correlation coefficient *r*=0·15 [95% CI –0·27 to 0·52]) but high correlation between Ag85A-specific IgG antibody responses on day 28 and peak IgG antibody responses on day 84 (*r*=0·67 [95% CI 0·39 to 0·83]) following MVA85A vaccination (appendix 3 p 8).

Previous sensitisation to vaccine antigens was seen in the IFN-γ ELISpot for Ag85A, PPD, and ChAd vector. Baseline IFN-γ ELISpot and IgG responses correlated positively with results on days 63 and 244 for Ag85A and PPD in both group 5 and group 6 (appendix 3 pp 7–8). Responses to the ChAd vector increased following vaccination in group 5, but returned to baseline by day 224 (appendix 3 p 13).

Exposure to *M tuberculosis* and acquisition of latent infection was monitored during the trials by including *M tuberculosis-*specific antigens ESAT-6/CFP-10 in ELISpot assays (appendix 3 p 13). Samples were considered ESAT-6/CFP-10 positive if average SFCs per 300 000 PBMCs was at least five SFCs more than double the negative control. For groups 1–4, conversion was noted in three (25%) of 12 participants: one (33%) of three participants from group 2 converted by day 28 and reverted by day 168, and two (67%) of three participants from group 1 converted on day 168, the last follow-up timepoint. In the randomised trial, we noted conversions in 14 (47%) of 30 participants in group 5 and 11 (38%) of 30 in group 6. Most reverted by the next visit, but one (3%) participant in group 5 and three (10%) participants in group 6 converted and remained IGRA-positive throughout the trial; none developed symptoms or signs of active tuberculosis.

## Discussion

Among Ugandan adolescents, ChAdOx1 85A was safe and immunogenic. As a booster to the BCG vaccine given at birth, ChAdOx1 85A–MVA85A induced stronger responses than BCG revaccination to vaccine antigen Ag85A, both in IFN-γ ELISpot and IgG ELISA assays; and similar responses to PPD. This was the first trial of the ChAdOx1 85A–MVA85A regimen in adolescents, and in a tuberculosis-endemic setting. The results support further development of viral-vector vaccines for tuberculosis. Further work is needed, including with multivalent vaccine candidates.

Although this was a small trial, and was not powered to detect rare SAEs, the results show no safety concerns in any group. AEs were mostly mild, there were no SAEs, and the four severe AEs each for ChAdOx1 85A and MVA85A were rapidly self-limiting. The AEs were similar to those described for viral-vectored vaccines.^24^ Although clinicians and participants were not masked to vaccine allocation, use of individualised diary cards reduced clinician bias of the safety assessment. Masking laboratory staff reduced bias in the immunogenicity assessment. Nesting of the trial within a long-standing birth cohort incurred potential bias in participant characteristics, but provided certainty as to BCG vaccination at birth, and opportunities, which we plan to exploit further, to explore effects of other life-course exposures on the vaccine responses.

Correlates of protection against tuberculosis remain poorly defined. Here we present results on both cellular and antibody responses, and to both the single vaccine antigen 85A and the complex antigens PPD and the BCG vaccine. The crucial role of IFN-γ is accepted,^25^ thus the strong ELISpot response to ChAdOx1 85A, further boosted by MVA85A, is promising. Serendipitously, due to the COVID-19 lockdown, we were able to observe the effect of delaying the MVA85A boost on the overall response and, reassuringly, found no effect. Current evidence suggests that antibodies are more important in protection against tuberculosis than previously thought.^15^ Most individuals showed strong Ag85A-specific IgG antibody responses to ChAdOx1 85A, boosted by MVA85A; few showed an Ag85A-specific IgG antibody response after BCG revaccination. Although ChAdOx1 85A–MVA85A contains a single mycobacterial antigen, the comparator vaccine, BCG, contains many, as does PPD, both including Ag85A. Our finding that ChAdOx1 85A–MVA85A induced responses similar to BCG revaccination for both PPD-specific IFN-γ ELISpot and PPD-specific IgG was therefore notable. However, BCG revaccination induced significantly higher BCG-specific ELISpot responses throughout follow-up than ChAdOx1 85A–MVA85A. BCG-specific CD4 T cells secreting IFN-γ were associated with reduced tuberculosis disease risk in South African infants, which might be important.^25^ As development of monovalent or multivalent viral vectored vaccines proceeds, trials comparing their efficacy with BCG revaccination will be important, accompanied by considerations of acceptability and feasibility (particularly if multiple doses are needed).

A long-standing concern in tuberculosis vaccine development is the decline in BCG efficacy with proximity to the equator,^7^ and whether new vaccines would be similarly affected. Observations for MVA85A suggest that this might be the case.^26^ Encouragingly, we found no statistically significant difference in ChAdOx1 85A–MVA85A response magnitude between the Oxford group^17^ and our Ugandan adolescents (appendix 3 pp 9, 15–16). However, this comparison is limited by sample size and differences in participants' age, and residents of urban Entebbe might be less exposed to factors that impair vaccine responses than rural, equatorial communities.

Individual responses to all three vaccines were very variable. Exposure to related viruses might interfere with the response to viral-vectored vaccines.^27^ Our trial was completed before children became eligible for COVID-19 vaccines, so pre-existing ChAd sensitisation was most likely due to cross-reactive natural exposures, rather than being vaccine-induced; however, we observed negative correlations between baseline ChAdOx1-GFP IFN-γ responses and day 63 and day 224 Ag85A-specific IFN-γ responses to ChAdOx1 85A–MVA85A (appendix 3 pp 9, 13), suggesting a possible adverse effect. Similarly, previous exposure to related environmental organisms has been proposed to mask the response to the BCG vaccine (by providing equivalent protection) or to block it (by interfering with induction of a response by the vaccine).^28^ Reassuringly, we found that baseline responses to Ag85A and PPD correlated positively with responses at follow-up, giving no suggestion of blocking. However, previous exposure to environmental mycobacteria might have been modest in urban Entebbe compared with rural settings and individuals with evidence of latent tuberculosis were excluded at baseline, although four participants developed sustained IGRA conversion during the follow-up period. Exposures to unrelated infections, or environmental, nutritional, or genetic factors might have important effects on the magnitude and durability of vaccine responses:^29^ we included participants irrespective of such exposures, so long as they were well (except for hepatitis B or C virus or HIV infection).

This study compared two approaches to boosting vaccine-induced immune responses to tuberculosis in the key target, adolescent age group in a tuberculosis-endemic setting. The IFN-γ ELISpot has been widely used in such trials as a measure of cellular responses. However, this assay most likely reflects effector responses rather than the recall long-lasting guardians of immunological memory, which include the memory natural killer cells, T cells, and B cells. Measurements of such recall response would be of interest as an indicator of possible longer-term protection, as would functional measures such as mycobacterial inhibition assays.^30^

In conclusion, our findings support the further development of booster vaccines against tuberculosis for adolescents using selected, and perhaps combined, mycobacterial antigens, and highlight the potentially valuable capacity of viral-vectored booster regimens to induce both cellular and antibody responses.

## Data sharing

De-identified participant data and study related documents can be accessed upon request from the corresponding author following publication of this Article.

## Declaration of interests

We declare no competing interests.

## Supplementary Materials

Swahili translation of the abstract

Luganda translation of the abstract
