# Supplementary material for: Safety and immunogenicity of ChAdOx1 85A prime followed by MVA85A boost compared with BCG revaccination among Ugandan adolescents who received BCG at birth: a randomised, open-label trial
Source: Lancet Infect Dis. 2024 Mar;24(3):285–96. doi: 10.1016/S1473-3099(23)00501-7 (PMC11876094; doi:10.1016/S1473-3099(23)00501-7)
Supplement: Swahili translation of the abstract [file mmc1.pdf]

THE LANCET
Infectious Diseases

Supplementary appendix 1

This translation in Swahili was submitted by the authors and we reproduce it as supplied. It has not been peer reviewed. *The Lancet*'s editorial processes have only been applied to the original in English, which should serve as reference for this manuscript.

Tafsiri hii katika Swahili iliwasilishwa na waandishi na tunatengeneza tena kama hutolewa. Haijapitiwa. Mchakato wa hariri wa Lancet Global Health umetumika tu kwa asili kwa Kiingereza, ambayo inapaswa kutumika kama kumbukumbu kwa muswada hii.

Supplement to: Wajja A, Nassanga B, Natukunda A, et al. Safety and immunogenicity of ChAdOx1 85A prime followed by MVA85A boost compared with BCG revaccination among Ugandan adolescents who received BCG at birth: a randomised, open-label trial. *Lancet Infect Dis* 2023; published online Nov 24. https://doi.org/10.1016/S1473-3099(23)00501-7.

Usalama na uwezo wa kinga ya mwili wa ChAdOx1 85A kuu ikifuatiwa na nyongeza ya MVA85A ikilinganishwa na kupata tena chanjo ya BCG miongoni mwa vijana waganda waliobalehe waliopata BCG wakati wa kuzaliwa: jaribio la nasibu, la kujitambulisha wazi

Muhtasari

Usuli BCG hutoa ulinzi uliopunguzwa, unaobadilika dhidi ya kifua kikuu cha mapafu. Chanjo yenye ufanisi zaidi inahitajika. Tulitathmini usalama na uwezo wa kingamwili wa kanuni ya watahiniwa ChAdOx1 85A–MVA85A ikilinganishwa na kupata tena chanjo ya BCG miongoni mwa vijana waganda waliobalehe.

Mbinu Baada ya ChAdOx1 85A kupanda kwa dozi na kupunguza umri, tulifanya jaribio la nasibu, la kujitambulisha wazi la awamu ya 2a kati ya vijana waliobalehe wenye afya nzuri wenye umri wa miaka 12-17, ambao walichanjwa BCG wakati wa kuzaliwa, bila ya kuambukizwa kifua kikuu, huko Entebbe, Uganda.

Washiriki waliteuliwa nasibu (1: 1) kwa kutumia ukubwa wa bloku ya saizi 6, kwa ChAdOx1 85A ikifuatiwa na MVA85A (siku ya 56) au BCG (mkazo wa Moscow). Wafanyikazi wa maabara walifunikwa kwa mgawo wa kikundi. Matokeo ya msingi yaliombwa na yasiyoombwa matukio mabaya (AEs) hadi siku ya 28 na matukio mabaya mabaya (SAEs), na majibu ya IFN-γ ELI Majibu ya antijeni

85A yalitazamwa (siku ya 63, na eneo chini ya mkunjo [AUC] siku 0-224) .

Matokeo Watu wazima sita (kikundi cha 1, n=3; kikundi cha 2, n=3) na vijana waliobalehe sita (kikundi cha 3, n=3; kikundi cha 4, n=3) waliandikishwa katika ongezeko la kipimo cha ChAdOx1 85A- pekee tafiti za kuongeza dozi na kupunguza umri

(Julai hadi Agosti, 2019). Katika jaribio la awamu ya 2a, vijana waliobalehe 60 waliteuliwa nasibu wakapewa ChAdOx1 85A–MVA85A bila mpangilio (kundi la 5, n=30) au BCG (kundi la 6, n=30; Desemba, 2019, hadi Oktoba, 2020). Washiriki wote 60 kutoka kwa vikundi 5 na 6 walijumuishwa katika uchanganuzi wa usalama, na 28 kati ya 30 kutoka kundi la 5 (ChAdOx1 85A–MVA85A) na 29 kati ya 30 kutoka kundi la 6 (walipata tena chanjo ya BCG) kuchambuliwa kwa matokeo ya kinga ya mwili. Katika jaribio la nasibu, AEs 60 ziliripotiwa kati ya 23 (77%) ya washiriki 30 kufuatia ChAdOx1 85A- MVA85A, 31 walikuwa wa kimfumo, na tukio moja kali baada ya kuongezeka kwa MVA85A ambalo lilijizuia haraka. Washiriki wote 30 katika kikundi kilichopata tena chanjo ya BCG waliripoti angalau AE moja ya wastani hadi ya wastani iliyoombwa; nyingi zilikuwa athari za ndani. Hakukuwa na SAEs katika vikundi vyote viwili. Majibu mahususi ya Ag85A IFN-γ ELI yalifikia kilele siku ya 63 katika kikundi cha ChAdOx1 85A– MVA85A na yalikuwa ya juu zaidi yakilinganishwa na kikundi cha urekebishaji chanjo ya BCG (uwiano wa kijiometri 30•59 [95% CI 17•46–53•59], p.

<0•0001, siku ya 63; tofauti ya wastani ya AUC 57 091 [95% CI 40 524–73 658], p<0•0001, siku 0–224).

Ufafanuzi Mfumo wa ChAdOx1 85A–MVA85A ulikuwa salama na ulisababisha majibu mahususi mahususi ya Ag85A kuliko kupata tena chanjo ya BCG. Matokeo yetu yanaunga mkono ukuzaji zaidi wa chanjo za kifua kikuu cha nyongeza.

Kufadhili Utafiti na Ubunifu wa Uingereza na Baraza la Utafiti wa Matibabu.
