# Supplementary material for: Safety and immunogenicity of ChAdOx1 85A prime followed by MVA85A boost compared with BCG revaccination among Ugandan adolescents who received BCG at birth: a randomised, open-label trial
Source: Lancet Infect Dis. 2024 Mar;24(3):285–96. doi: 10.1016/S1473-3099(23)00501-7 (PMC11876094; doi:10.1016/S1473-3099(23)00501-7)
Supplement: Luganda translation of the abstract [file mmc2.pdf]

THE LANCET
Infectious Diseases

Supplementary appendix 2

This translation in Luganda was submitted by the authors and we reproduce it as supplied. It has not been peer reviewed. The Lancet's editorial processes have only been applied to the original in English, which should serve as reference for this manuscript.

Ekiwandiiko kino ekikyuse mu lulimi Oluganda kyaweereddwayo abawandiisi b'ennyini era tukifulumizza nga bwe baakituwadde. Tekinnakubaganyizibwako birowoozo abakugu abalala. Okusunsula kwa Lancet kukoleddwa ku ekyo eky'Olungereza kyokka nga bwe kyafulumizibwa era kye kirina okujulizibwako ku kiwandiiko kino.

Supplement to: Wajja A, Nassanga B, Natukunda A, et al. Safety and immunogenicity of ChAdOx1 85A prime followed by MVA85A boost compared with BCG revaccination among Ugandan adolescents who received BCG at birth: a randomised, open-label trial. *Lancet Infect Dis* 2023; published online Nov 24. https://doi.org/10.1016/S1473-3099(23)00501-7.

Engeri eddagala lya ChAdOx1 85A gye litalina mutawaana n'engeri gye lisisimulamu obutaffaali obulwanyisa obuwuka mu mubiri nga liddirirwa eddagala lya MVA85A eryongereza ku bukuumi eri omubiri bw'ogeraageranya n'ekyokuddamu okugemebwa mu Bannayuganda abaakavubuka abaakubwa eddagala lya BCG nga baakazaalibwa: okugezesa ku balikozesa n'abakozesa ekirala.

Mu bufunze

Okunnyonnyola

BCG ewa obukuumi butono obukyukakyuka obw'obulwadde bw'akafuba obukosa amawuggwe. Eddagala eddala erisingako okugema obulungi lyetaagisa. Twekenneenya engeri eddagala lya ChAdOx1 85A–MVA85A gye lisisimulamu obutaffaali obulwanyisa obuwuka mu mubiri bw'ogeraageranya n'okuddamu okugemebwa ne BCG mu Bannayuganda abaakavubuka.

Enkola Oluvannyuma lw'okwongeza ku doozi ya ChAdOx1 85A n'okukendeeza ku myaka, twakola okugezesa ku balikozesa n'abakozesa ekirala ku mutendera gw'okugezesa ogwa 2a mu baana abaakavubuka abalamu obulungi abali wakati w'emyaka 12–17 egy'obukulu, abaagemebwa ne BCG nga baakazaalibwa, nga tebalinaako kabonero konna ak'obulwadde bw'akafuba Entebbe, mu Uganda. Abeetabi baalondebwanga awatali kigobererwa kyonna (1:1) nga bakozesa ennamba ey'awamu ya bantu 6, ku ChAdOx1 85A ne bazzaako MVA85A (ku lunaku olw'a 56) oba BCG (Ekika kya Moscow). Abakola mu laabu baabikkirirwa okubaako ne bye bakola mu kibinja. Ebyavaamu byali ebyo ebigwawo ebyali bisuubirwa n'ebyali bitasuubirwa kungaanyizibwa (AEs) okutuusa ku lunaku olwa 28 n'ebigwawo eby'obulabe ennyo (SAEs), n'enneeyisa eri IFN-γ ELISpot eri eddagala erirwanyisa obuwuka erya 85A (ku lunaku olw'e 63, n'ekifo ekyebunguluddwa [AUC] ennaku 0–224).

Ebyazuulibwa Abantu abakulu mukaaga (ekibinja 1, n=3; ekibinja 2, n=3) n'abaakavubuka mukaaga (ekibinja 3, n=3; ekibinja 4, n=3) baateekebwa ku ChAdOx1 85A- nga bagenda ku kunoonyereza okw'okwongezebwa kwa doozi n'okukendeeza ku myaka (Ogwomusanvu okutuuka mu Gwomunaana, 2019). Mu kugezesa okw'omutendera 2a, abaakavubuka 60 be baateekebwa ku ChAdOx1 85A–MVA85A (ekibinja 5, n=30) oba BCG (ekibinja 6, n=30; Ogwekkumineebiri, 2019, okutuuka mu mwezi Ogwekkumi, 2020). Bonna abeetabi 60 okuva mu kibinja eky'oku 5 n'o 6 baayingizibwa mu kwekenneenya oba nga teririna mutawaana nga abantu 28 ku 30 okuva mu kibinja eky'oku 5 (ChAdOx1 85A–MVA85A) n'abantu 29 ku 30 okuva mu kibinja 6 (BCG ab'okuddamu okugemebwa) beekenneenyezebwa ku biva mu kulaba oba nga eddagala teririna mutawaana. Mu kugezesa ku bantu abamaze galondebwa, AEs 60 ze zaazuulwa mu bantu 23 (77%) ku beetabi 30 okuddirirwa ChAdOx1 85A–MVA85A, 31 baali bukungi, nga waaliwo omu eyali mu mbeera embi oluvannyuma lw'okuweebwa MVA85A eryongera ku bukuumi nga ligenda limukugira mangu. Abeetabi bonna 30 mu kibinja kya BCG eky'okuddamu okugemebwa waakiri baalaga nti baalinayo AE nga ntono ddala; abasinga obungi baali balaga bubonero o wa bulijjo. Tewaaliwo SAEs mu bibinja byombiriri. Abaali ku Ag85A-lyokka IFN-γ ELISpot obubonero bwavaayo ku lunaku olw'e 63 mu kibinja kya ChAdOx1 85A– MVA85A era bwali wagguluko bw'ogeraageranya ku kibinja ky'okuddamu okugemebwa BCG (ekipimo kya ratio 30·59 [95% CI 17·46–53·59], p<0·0001, day 63; AUC n'enjawulo eya 57 091 [95% CI 40 524–73 658], p<0·0001, ennaku 0–224).

Entaputa Eddagala kya ChAdOx1 85A–MVA85A lyali teririna bulabe era nga lyaleetera Ag85A-okuzaala amaanyi amangi okusinga okuddamu okugemebwa BCG. Bye twazuula byongera okuwagira eky'okwongera okuvaayo n'eddagala ery'ongera amaanyi mu kugema obulwadde bw'akafuba.

Abataddemu ensimbi Akakiiko ka UK akavunaanyizibwa ku kunoonyereza n'obuvumbuzi n'akakiiko akanoonyereza ku by'obujjanjabi oba bayite UK Research and Innovations and Medical Research Council.
