# Supplementary material for: Safety and immunogenicity of ChAdOx1 85A prime followed by MVA85A boost compared with BCG revaccination among Ugandan adolescents who received BCG at birth: a randomised, open-label trial
Source: Lancet Infect Dis. 2024 Mar;24(3):285–96. doi: 10.1016/S1473-3099(23)00501-7 (PMC11876094; doi:10.1016/S1473-3099(23)00501-7)
Supplement: Supplementary appendix 3 [file mmc3.pdf]

THE LANCET
Infectious Diseases

Supplementary appendix 3

This appendix formed part of the original submission and has been peer reviewed. We post it as supplied by the authors.

Supplement to: Wajja A, Nassanga B, Natukunda A, et al. Safety and immunogenicity of ChAdOx1 85A prime followed by MVA85A boost compared with BCG revaccination among Ugandan adolescents who received BCG at birth: a randomised, open-label trial. *Lancet Infect Dis* 2023; published online Nov 24. https://doi.org/10.1016/S1473-3099(23)00501-7.

Safety and immunogenicity of ChAdOx1 85A prime followed by MVA85A boost compared to BCG revaccination among Ugandan adolescents who received BCG at birth: a randomised, open-label trial

Supplementary (Appendix) material

Content	*Page number*
Detailed laboratory methods	
Enzyme-Linked Immunospot (ELISpot) assay	*2*
Enzyme-linked immunosorbent assay (ELISA)	*2*
Malaria PCR	*2-3*
Helminths PCR	*3-4*
Appendix tables	
Table A1: Median time between ChAdOx1 85A and MVA85A boost (Group 5)	*5*
Table A2: Overall summary of reactogenicity and adverse events post vaccination for Groups 1 to 4	*5*
Table A3: Overall summary of reactogenicity and adverse events within 28 and 14 days of vaccination (Groups 5 and 6)	*6*
Table A4: Severity of reactogenicity and adverse events post vaccination (Groups 1 to 4), [Number of participants (total events)]	*6*
Table A5: Severity of reactogenicity and adverse events occurring within 28- and 14-days post vaccination (Groups 5 and 6), [Number of participants (total events)]	*7*
Table A6: Geometric mean IFN-γ ELISpot and IgG antibody responses to Ag85A and PPD at day 14 for Groups 1-4	*7*
Table A7: Correlation (Pearson's correlation coefficient) between vaccine responses and corresponding baseline responses among ChAdOx1 85A-MVA85A participants (Group 5)	*7*
Table A8: Correlation (Pearson's correlation coefficient) between vaccine responses and corresponding baseline responses among BCG participants (Group 6	*8*
Table A9: Correlation (Pearson's correlation coefficient) between IFN-γ responses and IgG responses among ChAdOx1 85A-MVA85A participants (Group 5)	*8*
Table A10: Correlation (Pearson's correlation coefficient) between IFN-γ responses and IgG responses among BCG participants (Group 6)	*8*
Table A11: Correlation (Pearson's correlation coefficient) between peak responses Ag85A-IFNy responses at day 63 and peak Ag85A-specific IgG responses at day 84 in the ChAdOx1 85A-MVA85A vaccination group (Group 5)	*8*
Table A12: Correlation (Pearson's correlation coefficient) between baseline ChAdOx1-GFP responses and Ag85A- specific IFN-γ and IgG responses at days 63 and 224 in groups 5 and 6	*9*
Table A13: IFN- γ and IgG responses to Ag85A in Ugandan adolescents (n=30) and UK adults (n=12) vaccinated with ChAdOx1 85A-MVA85A	*9*
Table A14: IFN- γ and IgG responses to Ag85A in Ugandan adolescents (n=7) and UK adults (n=12) in the ChAdOx1 85A-MVA85A trial arm, who received MVA85A at similar timepoints	*9*
Table A15: Comparison of Ag85A- and PPD- specific IFN-γ ELISpot and IgG responses in volunteers whose follow up time points post D28 were either on time or delayed due to COVID-19 pandemic	*10*
Appendix figures	
Figure A1: Ex vivo IFN-γ ELISpot responses to Ag85A, PPD, BCG, ChAdOx1-GFP, and ESAT-6/CFP-10 in Groups 1-4 participants vaccinated with ChAdOx1 85A	*11-12*
Figure A2: Plasma IgG responses to Ag85A and PPD in Groups 1-4 participants vaccinated with ChAdOx185A	*13*
Figure A3: Ex vivo IFN-γ ELISpot responses to ChAdOx1-GFP and ESAT-6/CFP-10 in adolescent volunteers vaccinated with ChAdOx1 85A-MVA85A and BCG revaccination	*13*
Figure A4: Comparison of Ag85A- and PPD- specific ex vivo IFN-γ ELISpot responses and IgG responses at D63 (or D372 for the delayed group) and D224 (or D533 for the delayed group) in volunteers whose follow up time points post D28 were either on time or delayed due to COVID-19 pandemic	*14*
Figure A5: IFN- γ ELISpot and plasma IgG responses to Ag85A in Ugandan adolescents (n=30) and UK adults (n=12) vaccinated with ChAdOx1 85A-MVA85A, irrespective of MVA85A time point administration.	*15*
Figure A6: IFN- γ ELISpot and plasma IgG responses to Ag85A in Ugandan adolescents (n=7) and UK adults (n=12) in the ChAdOx1 85A-MVA85A trial arm, who received MVA85A at similar timepoints.	*16*
References	*17*

Detailed laboratory methods

Enzyme-Linked Immunospot (ELISpot)

Ex-vivo IFN-γ ELISpot assays were performed on fresh peripheral mononuclear cells (PBMCs) collected from volunteers in Groups 1-4 at screening (day [D]0), D14, D28, D56 and D168. In Groups 5 and 6 this was done at screening (D0), D14, D28, D56, D63, D84, D140 and D224, as previously described.[1,2] Assays were done using human IFN-γ ELISpot (ALP) kits (Mabtech, Sweden). The PBMCs ($0.3x10^6$/well) were stimulated in triplicate with $2x10^5$ CFU/ml BCG (Serum Institute India), 20μg/ml Tuberculin PPD RT-23 (AJVaccines A/S, Denmark), and 2μg/ml a single pool of antigen 85A peptides (66 15-mer peptides, overlapping by 10 amino acids); 2μg/ml ESAT-6 and CFP-10 (pools of 15-mer peptides, Peptide Synthetics UK) were included to determine latent TB infection status; 2IU/1 PBMC ChAdOx1-GFP (Vector Core Facility, The Jenner Institute, Oxford, UK) was included to determine vaccine vector specific responses. Medium only wells were included as negative control, and wells stimulated with either 10μg/ml SEB (Sigma, UK) or a combination of 10μg/ml PHA (Sigma, UK) and 50ng/ml PMA (Sigma, UK) as positive controls. IFN-γ- spot forming cells (SFC) were enumerated using AID ELISpot reader (ELR08IFL, Autoimmun Diagnostika GMBH). ELISpot assays with more than 20 spots/well in the negative control wells and less than 200 spots/ well in the positive control wells were considered invalid and therefore repeated on frozen PBMCs. Mean background IFN-γ responses in the negative control wells were subtracted from mean IFN-γ responses in antigen stimulated, with responses reported as SFC/ million after correction for the number of PBMCs/ well ($0.3x10^6$). Samples with ESAT-6 and CFP-10 spot counts twice that in negative control and at least 5 spots more than the negative control wells were considered latent TB positive.

Enzyme-linked immunosorbent assay (ELISA)

ELISA was used to measure the Ag85A-specific and PPD-specific IgG antibody levels, as described elsewhere.[2] Cryopreserved plasma samples collected at similar timepoints as the PBMCs for the ELISpot assays, were used. Briefly, Immulon® 4 HBX microtitre plates (Thermo Scientific, USA) were coated with 50μl per well of 5μg/ml of recombinant Ag85A (BEI Resources, National Institute of Allergy and Infectious Diseases, USA) or 10μg/ml Tuberculin PPD RT23 (AJVaccines A/S, Denmark) in sodium bicarbonate buffer, and incubated overnight at 4°C. The plates were blocked for 2 hours at room temperature. 50μl of sample diluted 1 in 50 for Ag85A IgG assay and 1 in 100 for PPD IgG assay were added in duplicate to corresponding antigen coated wells and plates incubated overnight at 4°C. A positive control of pooled plasma samples from active TB patients was included per ELISA plate to test for plate to plate variability. Following overnight incubation, 50μl per well polyclonal anti-human IgG horseradish peroxidase (HRP; Dako, Denmark) detection antibody was added at a concentration of 0.5g/ml and plates were incubated for 1 hour at room temperature. 100μl per well o-phenylenediamine OPD (Sigma-Aldrich, USA) substrate for HRP was added and the plates were incubated for 15 minutes, in the dark, at room temperature. The reaction was stopped by adding 25μl per well of 2 M sulphuric acid. Absorbance in terms of optical density (OD) was measured at 490nm test wavelength and 630nm reference wavelength using ELISA plate reader (BioTek Instruments, USA). ODs for Ag85A-specific and PPD-specific IgG responses in test samples were obtained by subtracting mean ODs of duplicate blank wells from mean ODs of duplicate test samples.

Malaria PCR

Malaria PCR was carried out to detect *Plasmodium falciparum* genomic DNA in blood samples from volunteers prior to enrolment in the study (at screening time point), as previously described.[2] DNA was extracted from 500μl EDTA anti-coagulated whole blood using QIAamp 250 DNA Blood Mini Kit (QIAGEN, Germany) according to manufacturer's instructions. 2μl of sample DNA were then added to 23μl Master Mix containing 10μM concentration of each *P. falciparum* Reverse (Plas 171R 5'-AAC CCA AAG ACT TTG ATT TCT CAT AA-3') and Forward (PFal-F 5'CCG

ACT AGG TGT TGG ATG AAA GTG TTA A-3') primers, 25µM concentration of each Phocine Herpesvirus (PhHV) Reverse (PhHV-337as 5'-GCG GTT CCA AAC GTA CCA A-3') and Forward (PhHV-267s 5'-GGG CGA ATC ACA GAT TGA ATC-3') primers, 5µM *P. falciparum* probe (Pfal114-XS 5'CTT TCG AGG TGA CTT TTA GAT-3') and 25µM PhHV probe (PhHV-305tq 5'-TTT TTA TGT GTC CGC CAC CAT CTG GAT C-3') (Biolegio, Nijmegen, the Netherlands), 25mM MgCl2 (Sigma), HotStarTaq Master Mix (Qiagen) and Nuclease free water (Qiagen). The extracted DNA was quantified using a Nano drop 2000c. Applied Biosystems (ABI) MicroAmp 96-well reaction plates (cat. No 4346906) were used. Each PCR run consisted of 11 10-fold serial dilutions of DNA extracted from malaria positive (>1000mps/200WBCs confirmed on microscopy) sample, as positive controls and a negative control with nuclease free water in place of DNA. The serial dilutions also helped to allow setting a Cycle threshold (Ct) value cut off for the test samples. PhHV DNA was used as the internal control for the assay and it was added to the PCR Master Mix. All reactions were done in duplicate. ABI 7500 Fast Real Time PCR system were used for amplification, detection and analysis. The cycling conditions were: 15 min at 95ºC followed by 50 cycles of 15s at 95ºC, 30s at 60ºC, and 30s at 72ºC. Samples were considered *P. falciparum* positive if mean Ct value was <40.

Helminths PCR

Helminths PCR was carried out to detect *Schistosoma mansoni*, *Necator americanus*, and *Strongyloides stercolaris* genomic DNA in stool samples collected from volunteers prior to enrolment in the study, as previously described.[2] Ethanol-cryopreserved stool samples were centrifuged at 13,000rpm for 3 minutes to get rid of the ethanol. The stool pellet was lysed by treatment with 2% polyvinylpolypyrrolidone in 200µl of PBS (Sigma-Aldrich) for overnight at -20 °C, subsequent heating at 100°C for 10 minutes, addition of 200µl of ATL (Qiagen) and proteinase K (Qiagen) in a 9:1 ratio, heating overnight at 55°C followed by incubation in 400µl AL (Qiagen) buffer at 70°C for 10 minutes. The resultant DNA in pure ethanol was purified using QIAamp DNA Mini Kit (cat no 51306, Qiagen), according to manufacturer's instructions. The extracted DNA was quantified using a Nano drop 2000c. 4µl of sample DNA were then added to 21µl Master Mix containing 80nM concentration of each *S. stercolaris* Reverse (Stro-R1 5'-TGC CTC TGG ATA TTG CTC AGT TC-3') and Forward (Stro-F1 5'GAA TTC CAA GTA AAC GTA AGT CAT TAG C-3') primers, 300nM concentration of each *N. americanus* Reverse (Nec-R1 5'-ATA ACA GCG TGC ACA TGT TGC-3') and Forward (Nec-F1 5'CTG TTT GTC GAA CGG TAC TTG C-3') primers, 800nM concentration of each *S. mansoni* Reverse (Ssp124R 5'-TCC CGA GCG YGT ATA ATG TCA TTA-3') and Forward (Ssp48F 5'GGT CTA GAT GAC TTG ATY GAG ATG CT-3') primers, 100nM concentration of each Phocine Herpesvirus (PhHV) Reverse (PhHV-337as 5'-GCG GTT CCA AAC GTA CCA A-3') and Forward (PhHV-267s 5'-GGG CGA ATC ACA GAT TGA ATC-3') primers, 100nM *S. stercolaris* probe (Stro-4-TRBhq2 5'ACA CAC CGS CCG TCG CTG C-3'), 100nM *N. americanus* probe (Nec-2-FAM (MGB) 5'CTG TAC TAC GCA TTG TAT AC-3'), 80nM *S. mansoni* probe (Ssp78T-RT 5'TGG GTT GTG CTC GAG TCG TGG C-3'), and 100nM PhHV probe (PhHV-305tq 5'-TTT TTA TGT GTC CGC CAC CAT CTG GAT C-3') (Biolegio, Nijmegen, the Netherlands), 3.5mM MgCl2 (Sigma), 0.1mg/ml Bovine Serum Albumin (BSA), HotStarTaq Master Mix (Qiagen) and Nuclease free water (Qiagen). Applied Biosystems (ABI) MicroAmp 96-well reaction plates (cat. No 4346906) were used. Each PCR run consisted of 7 10-fold serial dilutions of a mixture of DNA from samples (from one of our studies) that were highly positive for *S. mansoni* and *N. americanus* on Kato-Katz, and DNA positive for *S. stercoralis* (kindly provided by Dr. Jaco J. Verweij, St. Elisabeth Hospital, Tilburg, the Netherlands). The serial dilutions were used to set a Cycle threshold (Ct) value cut off for the test samples. PhHV DNA was used as the internal control for the assay and it was added to the PCR Master Mix. Nuclease free water was used as a negative control. All reactions were done in duplicate. ABI 7500 Fast Real Time PCR system were used for amplification, detection and analysis. The cycling conditions were: 10 min at 95ºC followed by 50 cycles of 15s at 95ºC, 30s at 60ºC, and 30s at 72ºC. Samples were considered *S. mansoni, N. americanus, or S. stercolaris* positive if mean Ct value was <40.

Table A1: Median time between ChAdOx1 85A and MVA85A boost (Group 5)

Group	Median days (IQR)	Min, Max days
Overall (n=28) *	286 (170-295)	56, 297
Boost delayed group(n=21)	290 (286-296)	284, 297
Boost as scheduled group(n=7)	56 (56-57)	56, 57

IQR: interquartile range; Min: minimum; Max: maximum. * The time between MVA85A boost and primary outcome measurement was seven days for all participants who provided a sample for the primary outcome assessment

Table A2: Overall summary of reactogenicity and adverse events post vaccination for Groups 1 to 4

	Group 1<br>ChAdOx1 85A at 5 x10^{9} vp<br>n=3		Group 2<br>ChAdOx1 85A at 2.5 x10^{10} vp<br>n=3		Group 3<br>ChAdOx1 85A at 5 x10^{9} vp<br>n=3		Group 4<br>ChAdOx1 85A at 2.5 x10^{10} vp<br>n=3	
	All Event	Related Events[a]	All Events	Related Events[a]	All Events	Related Events[a]	All Events	Related Events[a]
Subjects with at least one solicited AE [n (%)]	3 (100%)	3 (100%)	1 (33%)	1 (33%)	3 (100%)	3 (100%)	3 (100%)	3 (100%)
Total # solicited AEs [n (# of AEs at severity grade 3)]	22 (1)	22 (1)	1(0)	1(0)	4(0)	4(0)	15(3)	15 (3)
Subjects with at least one solicited local AE	3 (100%)	3 (100%)	1 (33%)	1 (33%)	1 (33%)	1 (33%)	3 (100%)	3 (100%)
Total # local solicited AEs	7	7	1	1	2	2	3	3
Average # of local solicited AEs per participant experiencing local AEs[b]	2·3	2.3	1	1	2	2	1	1
Subjects with at least one solicited systemic AE	3	3	0	0	2	2	3	3
Total # systemic solicited AEs	15	15	0	0	2	2	12	12
Average # of solicited systemic AEs per participant experiencing systemic AEs[b]	5	5	-	-	1	1	4	4
Subjects with at least one unsolicited AE within 28 days of vaccination [n (%)]	1 (33%)	0 (0%)	1 (33·3%)	0 (0%)	0 (0%)	0 (0%)	1 (33·3%)	0 (0%)
Total # unsolicited AEs within 28 days of Vaccination [n (# of AEs at severity grade 3)]	2 (0)	0 (0)	1(0)	0 (0)	0 (0)	0 (0)	2 (0)	0 (0)
Subjects with an unsolicited severe AE [n (%)]	0 (0%)	0 (0%)	0 (0%)	0 (0%)	0 (0%)	0 (0%)	0 (0%)	0 (0%)
Subjects experiencing a SAE [n (%)]	0 (0%)	0 (0%)	0 (0%)	0 (0%)	0 (0%)	0 (0%)	0 (0%)	0 (0%)
Total # of SAEs[c]	0	0	0	0	0	0	0	0

AE: adverse event; SAE: serious adverse event. [a] all solicited events are considered to be at least possibly related to the study product. [b] Calculated as the number of AEs divided by the number of participants. [c] No serious event was recorded throughout the follow-up period

Table A3: Overall summary of reactogenicity and adverse events within 28 and 14 days of vaccination (Groups 5 and 6)

	Group 5 ChAdOx1 85A n=30		Group 5 MVA85A n=28		Group 6 BCG revaccination n=30	
	All Events	Related Events[a]	All Events	Related Events[a]	All Events	Related Events[a]
Subjects with at least one solicited AE [n (%)]	13 (43%)	13 (43%)	10 (36%)	10 (36%)	30 (100%)	30 (100%)
Total # solicited AEs [n ((# of AEs at severity grade 3)]	37 (0)	37 (0)	21(4)	21 (4)	82 (0)	82 (0)
Subjects with at least one solicited local AE	10	10	9	9	30	30
Total # local solicited AEs	16	16	11	11	68	68
Average # of local solicited AEs per participant experiencing local AEs[b]	1·6	1·6	1·2	1·2	2·3	2·3
Subjects with at least one solicited systemic AE	11	11	4	4	9	9
Total # systemic solicited AEs	21	21	10	10	14	14
Average # of solicited systemic AEs per participant experiencing systemic AEs[b]	1·9	1·9	2·5	2·5	1·6	1·6
Subjects with at least one unsolicited AE within 28 days of vaccination [n (%)]	2 (7%)	2 (7%)	1 (4%)	1 (4%)	4 (13%)	3 (10%)
Total # unsolicited AEs within 28 days of vaccination [n (# of AEs at severity grade 3)]	3 (0)	2 (0)	1 (0)	1 (0)	5 (0)	4 (0)
Subjects with an unsolicited severe AE [n (%)]	0 (0%)	0 (0%)	0 (0%)	0 (0%)	0 (0%)	0 (0%)
Subjects experiencing a SAE [n (%)]	0 (0%)	0 (0%)	0 (0%)	0 (0%)	0 (0%)	0 (0%)
Total # of SAEs[c]	0	0	0	0	0	0

AE: adverse event; SAE: serious adverse event. [a] all solicited events are considered to be at least possibly relate to the study product. [b] Calculated as the number of AEs divided by the number of participants. [c] No serious event was recorded throughout the follow-up period

Table A4: Severity of reactogenicity and adverse events post vaccination (Groups 1 to 4), [Number of participants (total events)]

	Group 1 ChAdOx1 85A at 5 x10^9 vp n=3			Group 2 ChAdOx1 85A at 2.5 x10^{10} vp n=3			Group 3 ChAdOx1 85A at 5 x10^9 vp n=3			Group 4 ChAdOx1 85A at 2.5 x10^{10} vp n=3		
Type of event	Mild	Moderate	Severe	Mild	Moderate	Severe	Mild	Moderate	Severe	Mild	Moderate	Severe
Solicited Local AEs	3 (5)	1 (2)	0 (0)	1 (1)	0 (0)	0 (0)	1 (2)	0 (0)	0 (0)	3 (3)	0 (0)	0 (0)
Solicited Systematic AEs	3 (12)	1 (2)	1 (1)	0 (0)	0 (0)	0 (0)	2 (2)	0 (0)	0 (0)	2 (7)	1 (2)	1 (3)
Unsolicited AEs	0 (0)	1 (2)	0 (0)	1 (1)	0 (0)	0 (0)	0 (0)	0 (0)	0 (0)	1 (2)	0 (0)	0 (0)

AE: adverse event. A participant can be counted twice i.e. reported a local/systemic adverse event of maximum grade 1 and a different local/systemic adverse event of maximum grade 2.

Table A5: Severity of reactogenicity and adverse events occurring within 28- and 14-days post vaccination (Groups 5 and 6), [Number of participants (total events)]

	Group 5 ChAdOx1 85A n=30			Group 5 MVA85A n=28			Group 6 BCG revaccination n=30		
**Type of event**	**Mild**	**Moderate**	**Severe**	**Mild**	**Moderate**	**Severe**	**Mild**	**Moderate**	**Severe**
Solicited Local AEs	9 (15)	1 (1)	0 (0)	7 (8)	1 (2)	1 (1)	30 (68)	0 (0)	0 (0)
Solicited Systematic AEs	11 (20)	1 (1)	0 (0)	3 (6)	1 (1)	1 (3)	8 (13)	1 (1)	0 (0)
Unsolicited AEs	2 (2)	2 (1)	0 (0)	1 (1)	0 (0)	0 (0)	3 (4)	1 (1)	0 (0)

AE: adverse event. A participant can be counted twice i.e. reported a local/systemic adverse event of maximum grade 1 and a different local/systemic adverse event of maximum grade 2.

Table A6: Geometric mean IFN-γ ELISpot and IgG antibody responses to Ag85A and PPD at day 14 for Groups 1-4

	**Dose escalation**		**Age de-escalation**	
	**Group 1 ChAdOx1 85A at 5 x10^{9} vp n=3**	**Group 2 ChAdOx1 85A at 2.5 x10^{10} vp n=3**	**Group 3 ChAdOx1 85A at 5 x10^{9} vp n=3**	**Group 4 ChAdOx1 85A at 2.5 x10^{10} vp n=3**
**Parameter**	**GM (SD)**	**GM (SD)**	**GM (SD)**	**GM (SD)**
**IFN-γ** Ag85A	135·04 (3·22)	141·94 (3·74)	592·97 (1·45)	440·64 (1·41)
**IFN-γ** PPD	164·66 (2·40)	130·94 (2·44)	239·42 (2·54)	197·20 (2·07)
IgG Ag85A	1·38 (1·11)	1·69 (1·13)	2·08 (1·20)	1·74 (1·10)
IgG PPD	1·60 (1·08)	1·90 (1·19)	1·70 (1·07)	1·73 (1·05)

GM: geometric mean; SD: standard deviation. SFC/million PBMC

Table A7: Correlation (Pearson's correlation coefficient) between vaccine responses and corresponding baseline responses among ChAdOx1 85A-MVA85A participants (Group 5)

	**Day 63**				**Day 224**			
**Baseline**	**IFN-γ-Ag85a**	**IFN-γ-PPD**	**IgG-Ag85a**	**IgG-PPD**	**IFN-γ-Ag85a**	**IFN-γ-PPD**	**IgG-Ag85a**	**IgG-PPD**
**IFN-γ-Ag85a**	0·35 (-0·03-0·65)				0·57 (0·26-0·78)			
**IFN-γ-PPD**		0·26 (-0·14-0·58)				0·48 (0·12-0·72)		
**IgG-Ag85a**			0·38 (0·01-0·66)				0·50 (0·15-0·73)	
**IgG-PPD**				0·76 (0·53-0·88)				0·75 (0·53-0·88)

Table A8: Correlation (Pearson's correlation coefficient) between vaccine responses and corresponding baseline responses among BCG participants (Group 6)

	Day 63				Day 224			
**Baseline**	**IFN-γ-Ag85a**	**IFN-γ-PPD**	**IgG-Ag85a**	**IgG-PPD**	**IFN-γ-Ag85a**	**IFN-γ-PPD**	**IgG-Ag85a**	**IgG-PPD**
**IFN-γ-Ag85a**	0·78 (0·56-0·90)				0·50 (0·16-0.74)			
**IFN-γ-PPD**		0·38 (-0·01-0·67)				0·05 (-0·33-0·41)		
**IgG-Ag85a**			0·79 (0·60-0·90)				0·89 (0·77-0·95))	
**IgG-PPD**				0·74 (0·51-0·87)				0·79 (0·59-0·90))

Table A9: Correlation (Pearson's correlation coefficient) between IFN-γ responses and IgG responses among ChAdOx1 85A-MVA85A participants (Group 5)

	Day 63				Day 224			
	**IFN-γ-Ag85a**	**IFN-γ-PPD**	**IgG-Ag85a**	**IgG-PPD**	**IFN-γ-Ag85a**	**IFN-γ-PPD**	**IgG-Ag85a**	**IgG-PPD**
**IFN-γ-Ag85a**			-0·03 (-0·40-0·36)	-0·22 (-0·55-0·18)			0·16 (-0·23-0·50)	-0·19 (-0·52-0·20)
**IFN-γ-PPD**			-0·09 (-0·45-0·30)	-0·32 (-0·62-0·07)			0·27 (-0·11-0·59)	-0·12 (-0·47-0·27)

Table A10: Correlation (Pearson's correlation coefficient) between IFN-γ responses and IgG responses among BCG participants (Group 6)

	Day 63				Day 224			
	**IFN-γ-Ag85a**	**IFN-γ-PPD**	**IgG-Ag85a**	**IgG-PPD**	**IFN-γ-Ag85a**	**IFN-γ-PPD**	**IgG-Ag85a**	**IgG-PPD**
**IFN-γ-Ag85a**			0·02 (-0·36-0·40)	0·37 (-0·02-0·66)			-0·14 (-0·48-0·24)	0·28 (-0·10-0·58)
**IFN-γ-PPD**			0·00 (-0·38-0·38)	0·31 (-0·08-0·62)			-0·20 (-0·53-0·18)	0·24 (-0·14-0·56)

Table A11: Correlation (Pearson's correlation coefficient) between peak responses Ag85A-IFNy responses at day 63 and peak Ag85A-specific IgG responses at day 84 in the ChAdOx1 85A-MVA85A vaccination group (Group 5)

	**IFN-γ-Ag85a-day63**	**IgG-Ag85a-day 84**
**IFN-γ-Ag85a-day 28**	0·15 (-0·27-0·52)	
**IgG-Ag85a-day 28**		0·67 (0·39-0·83)

Table A12: Correlation (Pearson's correlation coefficient) between baseline ChAdOx1-GFP responses and Ag85A- specific IFN-γ and IgG responses at days 63 and 224 in groups 5 and 6

	Group 5 ChAdOx1 85A-MVA85A				Group 6 BCG revaccination			
	Day 63		Day 224		Day 63		Day 224	
	IFN-γ-Ag85a	IgG-Ag85a	IFN-γ-Ag85a	IgG-Ag85a	IFN-γ-Ag85a	IgG-Ag85a	IFN-γ-Ag85a	IgG-Ag85a
**ChAd0xGFP baseline**	-0·08 (-0·45-0·31)	-0·03 (-0·40-0·35)	0·00 (-0·37-0·38)	-0·10 (-0·46-0·28)	0·12 (-0·29-0·49)	0·01 (-0·38-0·39)	0·16 (-0·24-0·51)	-0·02 (-0·40-0·36)

Table A13: IFN- γ and IgG responses to Ag85A in Ugandan adolescents (n=30) and UK adults (n=12) vaccinated with ChAdOx1 85A-MVA85A

	AUC (0-224 days)			Day 63							Day 224						
				Unadjusted				Adjusted*			Unadjusted				Adjusted*		
**Trial arm**	**n**	**Mean difference (AUC) (95% CI)**	**P value**	**n**	**GM†(SE)**	**GMR (95% CI)**	**P value**	**n**	**GMR (95% CI)**	**P value**	**n**	**GM†(SE)**	**GMR (95% CI)**	**P value**	**n**	**GMR (95% CI)**	**P value**
	**IFN-γ-Ag85a**																
UK	12	30940 (-1330-63211)	0·06	12	1128·18 (1·20)	1·45 (0·85-2·46)	0·17	12	1·38 (0·82-2·32)	0·22	12	263·99 (1·36)	1·18 (0·59-2·37)	0·63	12	1·07 (0·56-2·03)	0·85
Uganda	30	Ref.		27	779·30 (1·17)	Ref.		27	Ref.		28	223·56 (1·20)	Ref.		28	Ref.	
	**IgG-Ag85a**																
UK	12	64 (-16-143)	0·11	12	2·24 (1·17)	1·10 (0·84-1·43)	0·49	12	1·20 (0·90-1·60)	0·20	10	1·47 (1·09)	0·88 (0·72-1·07)	0·19	10	0·98 (0·80-1·19)	0·82
Uganda	30	Ref.		28	2·04 (1·06)	Ref.		28	Ref.		28	1·68 (1·05)	Ref.		28	Ref.	

AUC: area under the curve; CI: confidence interval; GM: geometric mean; SE: standard error; GMR: geometric mean ratio. *Adjusted for corresponding baseline responses

Table A14: IFN- γ and IgG responses to Ag85A in Ugandan adolescents (n=7) and UK adults (n=12) in the ChAdOx1 85A-MVA85A trial arm, who received MVA85A at similar timepoints

	AUC (0-224 days)			Day 63							Day 224						
				Unadjusted				Adjusted*			Unadjusted				Adjusted*		
**Trial arm**	**n**	**Mean difference (AUC) (95% CI)**	**P value**	**n**	**GM†(SE)**	**GMR (95% CI)**	**P value**	**n**	**GMR (95% CI)**	**P value**	**n**	**GM†(SE)**	**GMR (95% CI)**	**P value**	**n**	**GMR (95% CI)**	**P value**
	**IFN-γ-Ag85a**																
UK	12	22786 (-26837-72409)	0·35	12	1128·18 (1·20)	1·04 (0·57-1·88)	0·90	12	1·02 (0·54-1·91)	0·96	12	263·99 (1·36)	1·22 (0·42-3·53)	0·70	12	1·39 (0·52-3·72)	0·49
Uganda	7	Ref.		6	1089·42 (1·14)	Ref.		6	Ref.		7	216·29 (1·48)	Ref.		7	Ref.	
	**IgG-Ag85a**																
UK	12	66 (-57-189)	0·27	12	2·24 (1·17)	0·96 (0·59-1·57)	0·87	12	1·01 (0·54-1·88)	0·97	10	1·47 (1·09)	0·90 (0·67-1·20)	0·45	10	1·11 (0·77-1·60)	0·55
Uganda	7	Ref.		7	2·33 (1·15)	Ref.		7	Ref.		7	1·63 (1·11)	Ref.		7	Ref.	

AUC: area under the curve; CI: confidence interval; GM: geometric mean; SE: standard error; GMR: geometric mean ratio *Adjusted for corresponding baseline responses

Table A15: Comparison of Ag85A- and PPD- specific IFN-γ ELISpot and IgG responses in volunteers whose follow up time points post D28 were either on time or delayed due to COVID-19 pandemic

ChAdOx1 85A-MVA85A					
		**n**	**Day 63**	**n**	**Day 224**
			**GMR (95% CI)**		**GMR (95% CI)**
**IFN-γ Ag85A**	No delay	6	1·54 (0·73-3·26)	7	0·96 (0·39-2·32)
	Delay	21	Ref.	21	Ref.
**IFN-γ PPD**	No delay	6	0·76 (0·41-1·41)	7	0·78 (0·25-2·42)
	Delay	21	Ref.	21	Ref.
**IgG Ag85a**	No delay	7	1·19 (0·92-1·55)	7	0·97 (0·76-1·23)
	Delay	21	Ref.	21	Ref.
**IgG PPD**	No delay	7	1·10 (0·92-1·30)	7	0·96 (0·83-1·12)
	Delay	21	Ref.	21	Ref.
**BCG revaccination**					
**IFN-γ Ag85A**	No delay	9	1·23 (0·39-3·93)	9	0·98 (0·26-3·66)
	Delay	18	Ref.	20	Ref.
**IFN-γ PPD**	No delay	9	1·51 (0·92-2·48)	9	1·17 (0·38-3·69)
	Delay	18	Ref.	20	Ref.
**IgG Ag85a**	No delay	9	0·94 (0·77-1·16)	9	0·97 (0·82-1·14)
	Delay	20	Ref.	20	Ref.
**IgG PPD**	No delay	9	1·07 (0·89-1·28)	9	1·02 (0·85-1·21)
	Delay	20	Ref.	20	Ref.

Appendix Figures

Figure A1: Ex vivo IFN-γ ELISpot responses to Ag85A, PPD, BCG, ChAdOx1-GFP, and ESAT-6/CFP-10 in Groups 1-4 participants vaccinated with ChAdOx1 85A

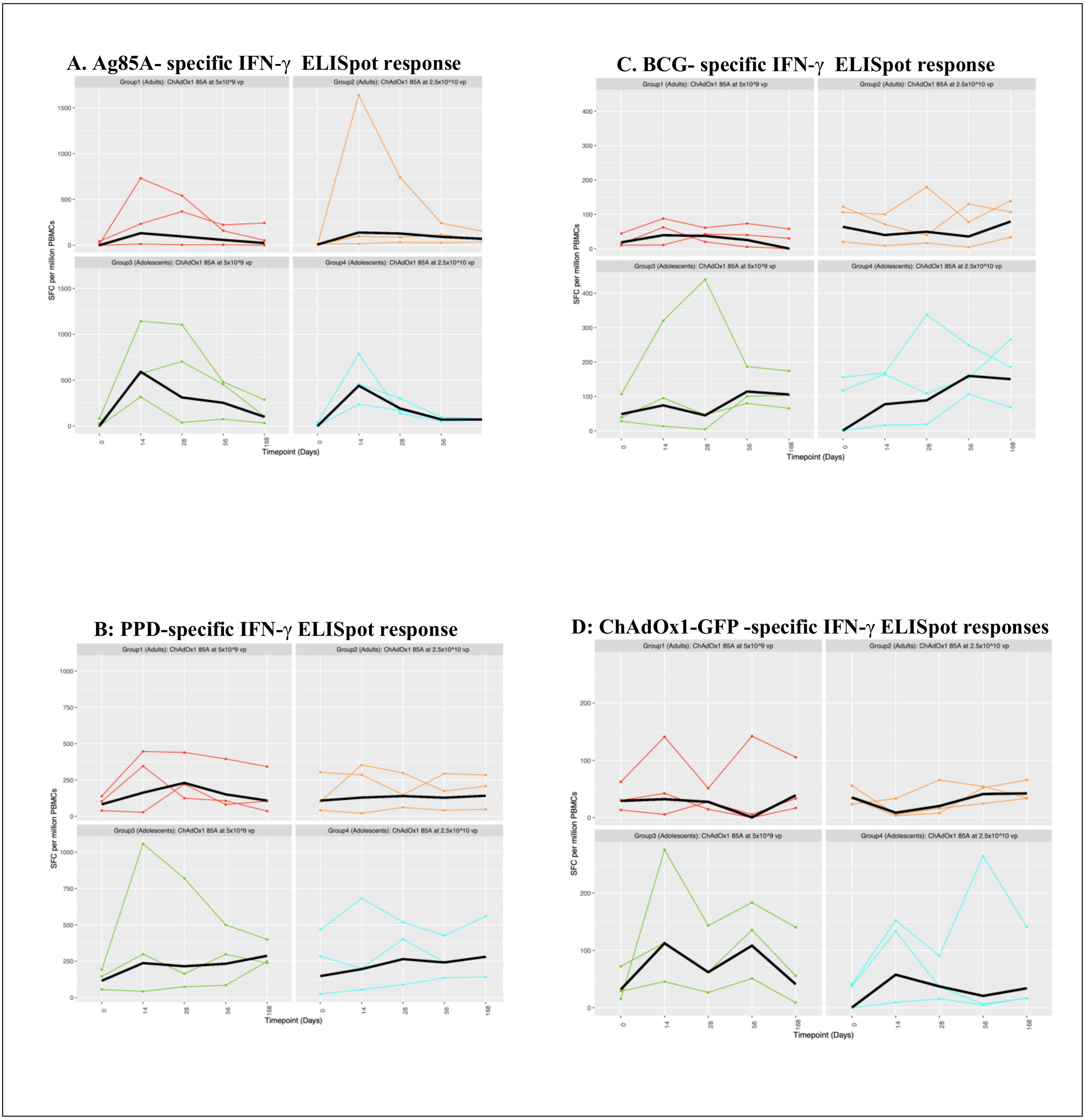

E. ESAT-6/CFP-10- specific IFN-γ ELISpot response

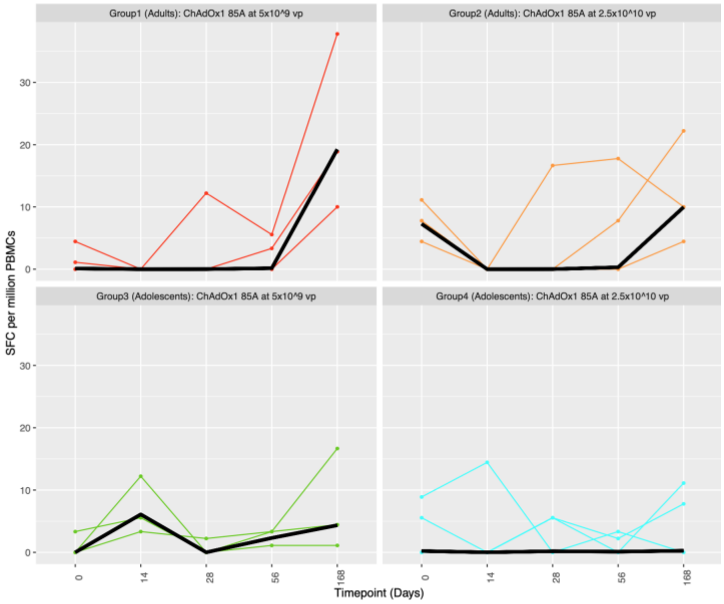

Figure A1: Ex vivo IFN-γ ELISpot responses (Spot Forming cells (SFC)/ $1x10^6$ PBMC) to Ag85A pool of 66 peptides, PPD, BCG, ChAdOx1-GFP, and ESAT-6/CFP-10 in Groups 1-4 participants vaccinated with ChAdOx1 85A at D0. Individual values are shown for each volunteer at each follow up timepoint. The black bold line represents geometric mean.

Figure A2: Plasma IgG responses to Ag85A and PPD in Groups 1-4 participants vaccinated with ChAdOx185A

A. Plasma IgG responses to Ag85A

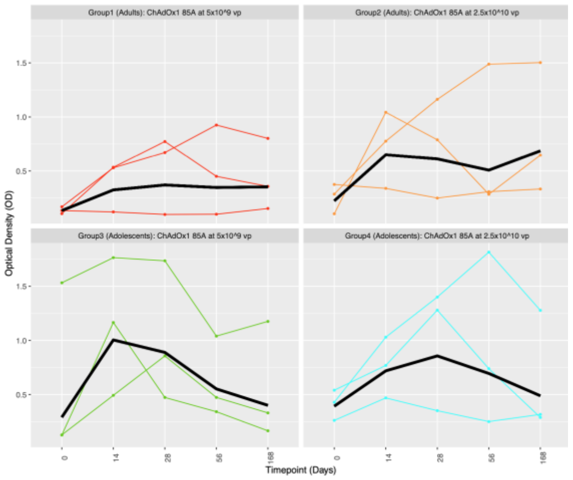

B. Plasma IgG responses to PPD

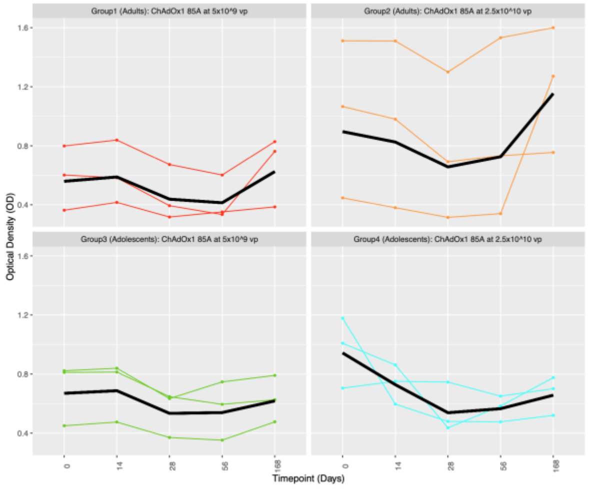

Figure A2: Plasma IgG responses to recombinant Ag85A and PPD in Groups 1-4 participants vaccinated with ChAdOx1 85A at D0. Individual values are shown for each volunteer at each follow up timepoint. The black bold line represents geometric mean.

Figure A3: Ex vivo IFN-γ ELISpot responses to ChAdOx1-GFP and ESAT-6/CFP-10 in adolescent volunteers vaccinated with ChAdOx1 85A-MVA85A and BCG revaccination.

A. ChAdOx1-GFP- specific IFN-γ ELISpot response

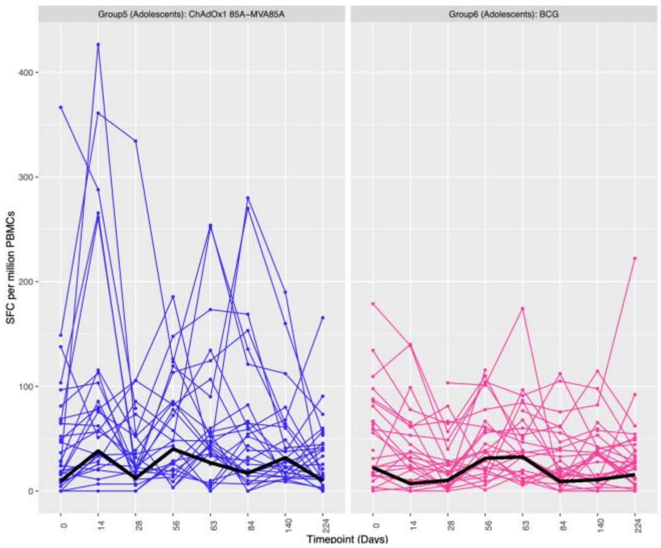

B. ESAT-6/CFP-10 - specific IFN-γ ELISpot response

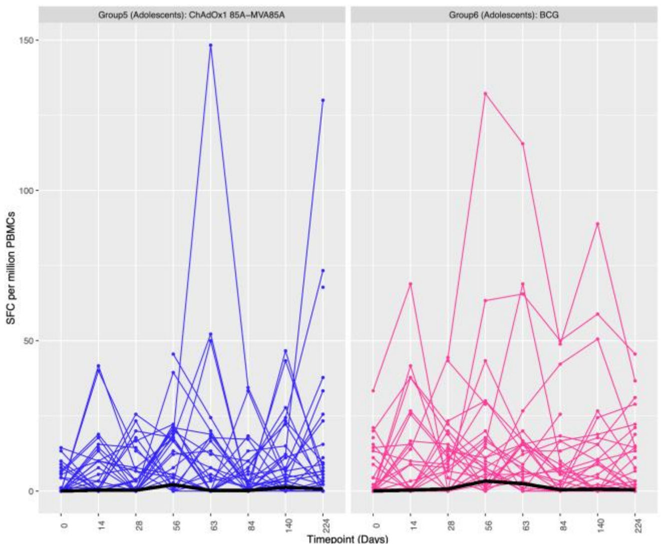

Figure A3. Ex vivo IFN-γ ELISpot responses to ChAdOx1-GFP and ESAT-6/CFP-10 in adolescent volunteers vaccinated with ChAdOx1 85A-MVA85A (Group 5) and BCG revaccination (Group 6). Individual values are shown for each volunteer at each follow up timepoint. The black bold line represents geometric mean. Follow up timepoints D56, D63, D84, D140 and D224 refer to D56 (or D365 for delayed group), D63 (or D372 for the delayed group), D84 (or D393 for the delayed group), D140 (D449 for the delayed group), and D224 (or D533 for the delayed group)

Figure A4

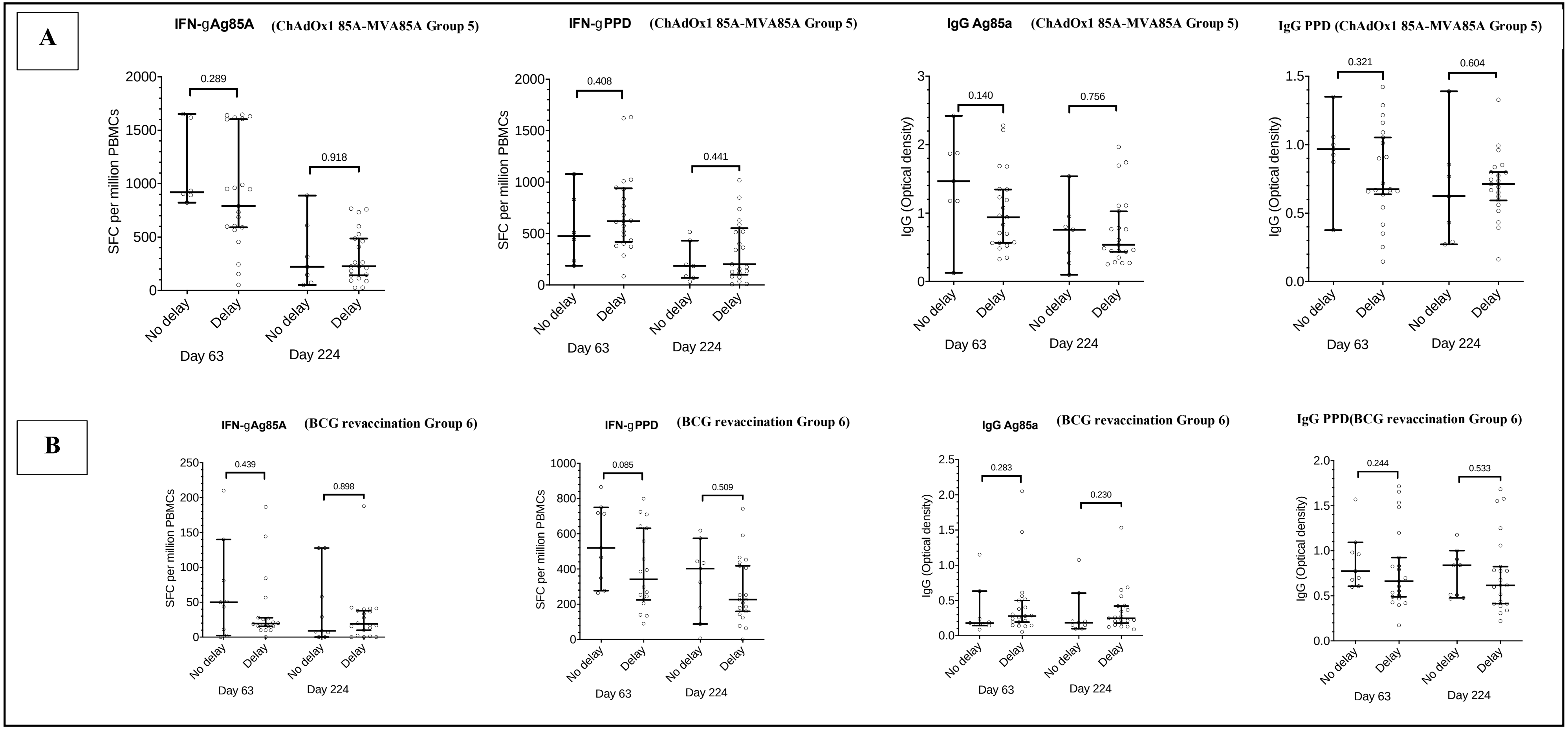

Figure A4: Comparison of Ag85A- and PPD- specific *ex vivo* IFN-γ ELISpot responses and IgG responses at D63 (or D372 for the delayed group) and D224 (or D533 for the delayed group) in volunteers whose follow up time points post D28 were either on time or delayed due to COVID-19 pandemic. Panels A and B represent *ex vivo* IFN-γ ELISpot responses and IgG responses in the ChAdOx1 85A-MVA85A and BCG revaccination group respectively. Individual values are shown for each volunteer. Median and 95% confidence interval are shown per group. Mann-Whitney test was used to compare the responses between on time and delayed group.

Figure A5

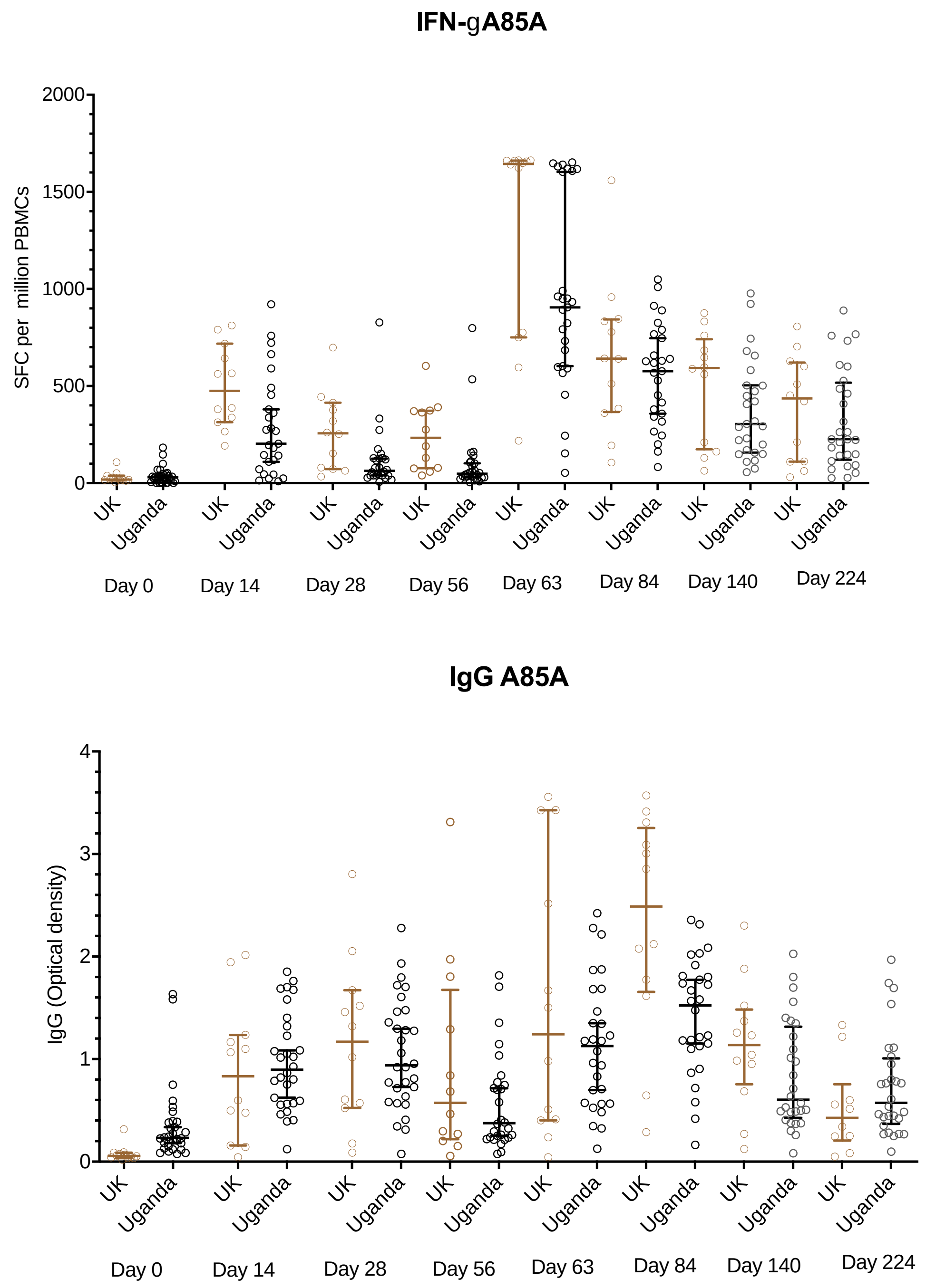

Figure A5: IFN- γ ELISpot and plasma IgG responses to Ag85A in Ugandan adolescents (n=30) and UK adults (n=12) vaccinated with ChAdOx1 85A-MVA85A, irrespective of MVA85A time point administration. Individual values are shown for each volunteer. Median and 95% confidence interval are shown per group. Mann-Whitney test was used to compare the responses between on Uganda and UK population.

Figure A6

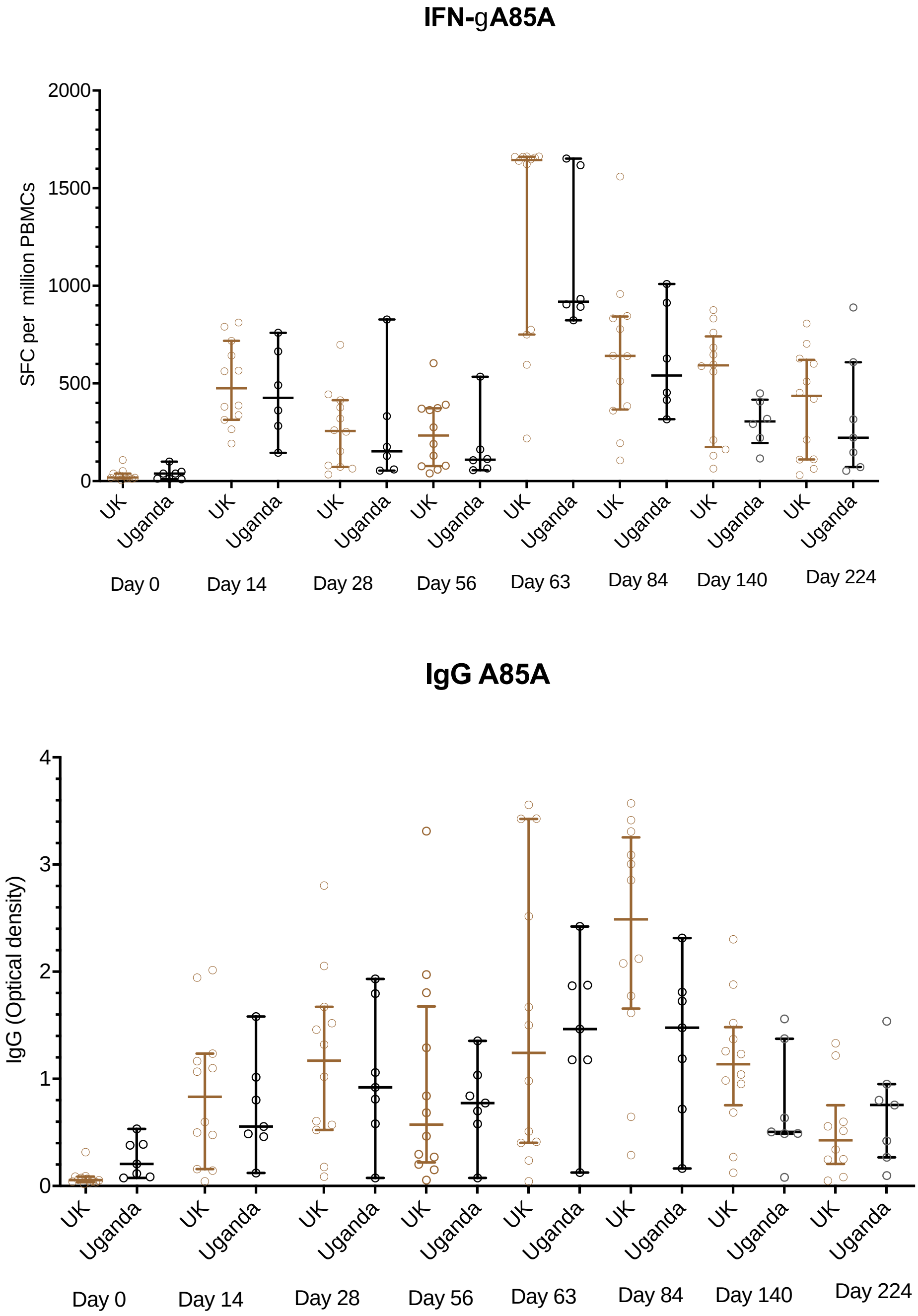

Figure A6: IFN- γ ELISpot and plasma IgG responses to Ag85A in Ugandan adolescents (n=7) and UK adults (n=12) in the ChAdOx1 85A-MVA85A trial arm, who received MVA85A at similar timepoints. Individual values are shown for each volunteer. Median and 95% confidence interval are shown per group. Mann-Whitney test was used to compare the responses between on Uganda and UK population.

References

1. Wilkie M, Satti I, Minhinnick A, Harris S, Riste M, Ramon RL, et al. A phase I trial evaluating the safety and immunogenicity of a candidate tuberculosis vaccination regimen, ChAdOx1 85A prime - MVA85A boost in healthy UK adults. Vaccine [Internet]. 2019/11/15. 2020 Jan 22;38(4):779–89. Available from: https://pubmed.ncbi.nlm.nih.gov/31735500

2. Wajja A, Kizito D, Nassanga B, Nalwoga A, Kabagenyi J, Kimuda S, et al. The effect of current Schistosoma mansoni infection on the immunogenicity of a candidate TB vaccine, MVA85A, in BCG-vaccinated adolescents: An open-label trial. PLoS Negl Trop Dis. 2017;11(5).
